# Discovery of Novel Plant Interaction Determinants from the Genomes of 163 Root Nodule Bacteria

**DOI:** 10.1038/srep16825

**Published:** 2015-11-20

**Authors:** Rekha Seshadri, Wayne G. Reeve, Julie K. Ardley, Kristin Tennessen, Tanja Woyke, Nikos C. Kyrpides, Natalia N. Ivanova

**Affiliations:** 1 Department of Energy Joint Genome Institute, Walnut Creek, USA; 2 Centre for Rhizobium Studies, School of Veterinary and Life Sciences, Murdoch University, Murdoch 6150, Australia; 3 Department of Biological Sciences, King Abdulaziz University, Jeddah, Saudia Arabia

## Abstract

Root nodule bacteria (RNB) or “rhizobia” are a type of plant growth promoting bacteria, typified by their ability to fix nitrogen for their plant host, fixing nearly 65% of the nitrogen currently utilized in sustainable agricultural production of legume crops and pastures. In this study, we sequenced the genomes of 110 RNB from diverse hosts and biogeographical regions, and undertook a global exploration of all available RNB genera with the aim of identifying novel genetic determinants of symbiotic association and plant growth promotion. Specifically, we performed a subtractive comparative analysis with non-RNB genomes, employed relevant transcriptomic data, and leveraged phylogenetic distribution patterns and sequence signatures based on known precepts of symbiotic- and host-microbe interactions. A total of 184 protein families were delineated, including known factors for nodulation and nitrogen fixation, and candidates with previously unexplored functions, for which a role in host-interaction, -regulation, biocontrol, and more, could be posited. These analyses expand our knowledge of the RNB purview and provide novel targets for strain improvement in the ultimate quest to enhance plant productivity and agricultural sustainability.

The use of plant growth promoting bacteria to enhance crop yield and control disease is gaining worldwide acceptance as a sustainable agricultural practice, while reducing costs by supplanting the use of expensive (and polluting) agrochemicals. These bacteria can facilitate plant growth either directly, by providing essential nutrients (nitrogen, phosphorus and essential minerals), modulating plant hormones and development, or indirectly, by suppressing inhibitory effects of various plant pathogens, improving soil structure and bioremediating polluted soils^1^,^2^. In particular, root nodule bacteria (RNB) are free-living soil bacteria that have the ability to form nitrogen-fixing symbioses with legumes, and have been exploited for centuries to improve soil fertility and agricultural productivity^3^. The symbiosis is typically host-specific (although more promiscuous strains exist) and mediated by signaling molecules produced by both plant host and the bacterium^4^. RNB convert inert atmospheric nitrogen gas into bioavailable ammonia for their host in exchange for carbon (and shelter) within specialized root or stem nodules, resulting in improved plant growth and productivity^5^.

The legume-RNB symbiosis is one of the best-studied associations between bacteria and eukarya due to both ecological and economic importance. It is estimated that increasing the efficiency of symbiotic nitrogen fixation (SNF) may have an annual benefit of $1,067 million in the U.S alone, while total elimination of nitrogen fertilization of major crops would have an annual benefit of $4,484 million^6^. Additionally, SNF reduces greenhouse gas emissions by displacing 873 m^3^ of natural gas and the ultimate release of ~2 tons of CO_2_^7^ in the manufacture every ton of conventional nitrogenous fertilizer, as well as reducing annual nitrous oxide emissions and NO_3_^−^ in surface runoff. Other benefits to the environment include reducing dryland salinity, increasing soil fertility, promoting carbon sequestration and preventing eutrophication of water bodies. Furthermore, RNB play a role in the production of biofuel crops–*Millettia pinnata*, for example, is a leguminous tree nodulated by *Bradyrhizobium* and *Rhizobium* spp. that produces biodiesel, starch, ethanol and biogas^8^. With a burgeoning world population and increasing food demands, harnessing the innate potential of RNB to improve sustainable agricultural productivity is of paramount importance.

However, despite these significant environmental and economic incentives, only a few genomes of a phylogenetically restricted group of model RNB strains had been sequenced at the inception of this study. These strains were mostly laboratory “work horses”, whereas sequencing of commercial inoculants that have the highly prized attributes of survival and persistence in soil, competitiveness, and high rates of N_2_-fixation, had not been a priority. In addition, preliminary analyses focused on questions pertaining to genome evolution and structure, intra-genus conservation and physiological diversity^9^,^10^,^11^. A more recent paper surveyed the occurrence of **known** plant growth promotion genes in all available proteobacterial genomes^12^, and clearly many RNB were found to possess plant growth promotion traits beyond nitrogen fixation, but little had been done to explore novel effectors of plant growth or even the accessory factors mediating RNB-plant interactions (including symbiosis). Thus, the primary objective of our study was to (i) increase the repertoire of available RNB genomes in terms of their phylogenetic, biogeographic and host legume diversity, and (ii) identify novel microbial effectors of symbiosis, and plant growth and productivity, beyond what is currently known about nodulation and nitrogen fixation. We sequenced the genomes of 110 RNB isolates sourced from a variety of leguminous hosts from diverse biogeographical locations, performed a comprehensive analysis of all RNB genera, and identified novel determinants of plant interaction and growth. These data not only provide a resource and conceptual framework for studying RNB-legume interactions, but our results highlight many new potential plant beneficial genes that could be targeted to improve legume productivity around the globe.

## Results and Discussion

### Overview of the Project

**Figure 1:**
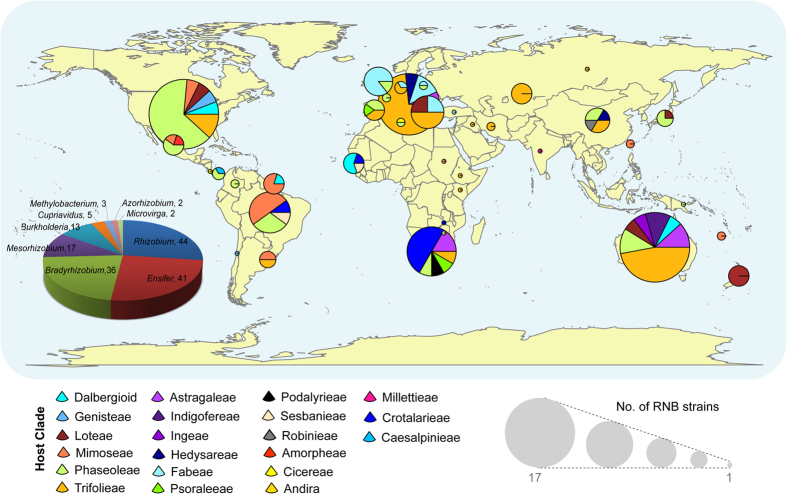
Summary of biogeography and taxonomy of 163 RNB strains analyzed in this study. Biogeographic information is depicted as world map overlaid with pie charts showing the legume clade of the plant host for strains originating from that geographic location; size of the pie scales to the total number of RNB strains from that location (ranging from 1 to 17 strains). Taxonomic composition of 163 RNB strains is shown as a separate pie chart in the bottom left side of the figure. See Supplementary Tables 1 and 2 for genome statistics and metadata details. Figure was generated using the R package “maps” (Brownrigg, R., Minka, T. P., Becker, R. A. & Wilks, A. R. maps: Draw Geographical Maps. R package version 2.1-5. http://CRAN.R-project.org/package=maps (2010), and edited further using Adobe Illustrator.

This study falls under the auspices of the Genomic Encyclopedia of Bacteria and Archaea (GEBA) project, which was conceived to maximize the phylogenetic coverage of publicly available prokaryotic genomes^13^,^14^,^15^. Correspondingly, the GEBA-RNB sub-project was designed to capture RNB phylogenetic and symbiotic diversity, with the participation of an international consortium consisting of more than 30 experts in the field, from 15 different countries, and major culture collection centers in Australia, Belgium and the USA^14^. RNB strains were selected on the basis of (i) phylogenetic diversity, (ii) host legume diversity (spanning all the Vavilov centers of origin^16^) (iii) economic or commercial significance and, (iv) biogeographic origin (Fig. 1). Strains were also required to have comprehensive experimental and metadata records and well-characterized phenotypes, in particular, relating to symbiotic efficiency and host specificity. Biogeographic considerations were relevant as RNB survival and persistence as soil saprophytes is governed by environmental and edaphic constraints such as pH, temperature, salinity, soil moisture- and clay content. The RNB were therefore collected from sites that spanned a broad range of soils (varying pH, salinity) and climates (e.g. tropical, arid, temperate). Chosen RNB also varied in their physiological traits (e.g., ability to recycle hydrogen, methylotrophy, salt or acid tolerance, rhizobitoxine production, heavy metal resistance, etc.) and host specificities (ranging from strictly specific to highly promiscuous). Moreover, each sequenced RNB strain has been cryopreserved in a dedicated long term storage culture collection and is available to the global research community by request through the Centre for Rhizobium Studies (CRS).

To summarize, 110 RNB isolates from 70 diverse legume hosts from various biomes in over 30 countries were sequenced by us, and an additional 50 genomes were released to Genbank during the course of this study, resulting in a total of 163 RNB genomes analyzed here (Fig. 1). All major RNB lineages were represented with the overwhelming majority (145 genomes) belonging to seven genera within the Order Rhizobiales of Class α-proteobacteria, and 18 genomes belong to two genera from Class β-proteobacteria. The complete list of RNB genomes, metrics and metadata is presented in Supplementary Table 1. General assembly and annotation metrics are presented in Supplementary Table 2.

### Predicting Novel Effectors of RNB-plant interaction

The RNB-legume symbiosis has been long-heralded as an excellent model for investigating plant-microbe associations; however, few studies have attempted to venture beyond describing the mechanisms and underpinnings of nodulation and N_2_-fixation, the hallmark ability of RNB. With few exceptions, auxiliary functions that are undoubtedly necessary to colonize, communicate or interact with their plant host, and possibly regulate plant development, are largely anonymous. It is also evident that many RNB possess capacities well beyond bio-fertilization through N_2_-fixation - for example, 1-aminocyclopropane-1-carboxylate (ACC) deaminase (TIGR01274), known to modulate plant development by reducing levels of the plant stress hormone, ethylene^17^, is almost ubiquitously present in all the sequenced RNB genera with the exception of several strains of *Ensifer* spp. and *Rhizobium* spp. Furthermore, the introduction of an exogenous ACC deaminase gene into the laboratory strain *Ensifer meliloti* Rm 1021, which lacks this gene, increased biomass and nodulation of host *Medicago sativa* (alfalfa or Lucerne) by over 40%^18^.

**Figure 2:**
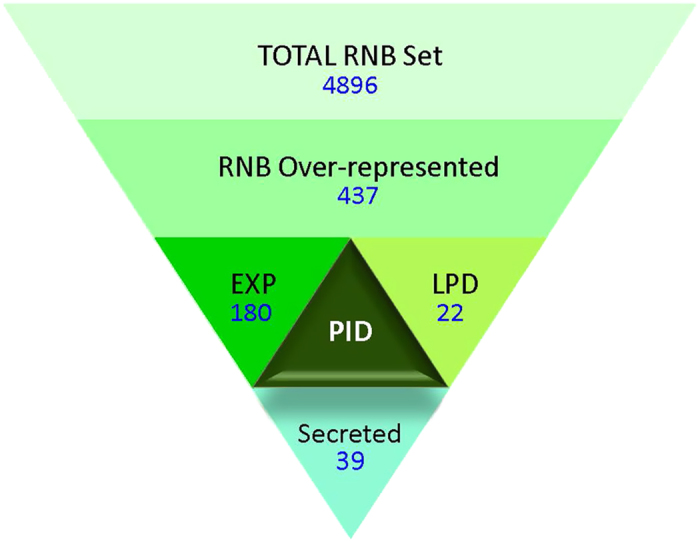
Delineating novel plant interaction determinants from 163 RNB genomes. Overall strategy devised to filter and identify protein family domains associated with plant interaction or growth promotion from 163 RNB genomes. LPD – limited phylogenetic distribution, EXP - upregulation or induction in published transcriptomic studies^21^,^22^,^23^,^24^, PID – plant interaction determinants, “Secreted” refers to Pfam candidates that bear a signal peptide for possible secretion into the external milieu. See Supplementary Table 4 for list with details.

A multi-step strategy was therefore devised to help identify novel plant-beneficial determinants from the 163 RNB genomes (Fig. 2), involving a subtractive comparative analysis with non-RNB genomes, leveraging relevant transcriptomic data for substantiation, and employing additional filters such as phylogenetic occurrence and sequence signatures based on known precepts of symbiotic and plant-microbe interactions. The first step identified functions that are over-represented or enriched in the RNB genomes set compared to a “negative control” (NC) genome set. To minimize the identification of false positives, the NC members were carefully selected from available genomes of phylogenetically-related organisms that are not known to be associated with the phytosphere (e.g., rhizosphere, phyllosphere) environment (based on available metadata for genomes from the GOLD database^19^). This resulted in a NC set containing 69 genomes from 35 genera belonging to either Order Rhizobiales of Class α-proteobacteria, or Class β-proteobacteria (Supplementary Table 3). To the best of our knowledge, these 69 isolates originated from a variety of aquatic, terrestrial and few host-associated habitats, and are not typically associated with the phytosphere, based on available GOLD metadata entries and published literature.

Next, gene counts for each protein family (Pfam) domain were retrieved and contrasted between the RNB and NC genomes. Pfam was chosen for this analysis because it is the largest and most widely used collection of manually-curated protein families^20^, with >80% coverage (on average) of total CDS in these microbial genomes.

A primary approach to identify Pfams that were “over-represented” in the RNB involved contrasting median or upper and lower quartile gene counts between the RNB and NC genomes. A total of 437 Pfams (out of 4896 Pfam domains recruited by 163 RNB genomes) could be delineated based on a total RNB median (2^nd^ quartile) gene count of ≥1 and a total NC median gene count of 0 (Supplementary Table 4). Encouragingly, many core nodulation and nitrogen fixation-related Pfams (e.g., NodA (PF02474), NifD (PF00148), NifK (PF11844)) were identified and served to validate our approach (core components not represented could be attributed to the absence of a suitable or specific Pfam domain for that particular function (e.g., NifH)). For many others, a role in plant host interaction, biocontrol, stress tolerance, or more could be posited (discussed below), however, this approach also yielded 123 Pfams containing domains of unknown function (DUFs), which may also be important in legume-RNB interactions.

To garner further support for a proposed role for these over-represented Pfams in phytosphere interactions, corroboration by transcript expression (designated “EXP”) under relevant experimental conditions was used as a key filtering step (Fig. 2). Out of 437 over-represented Pfams, 180 had a single candidate gene that showed upregulation or induction in one or more of four published RNB transcriptome studies of symbiotic nitrogen fixation^21^,^22^,^23^,^24^. For example, PhoD-like phosphatase family (PF09423) candidates were induced >64X in the symbiosome, in two independent RNA-seq-based transcriptome studies (*Ensifer fredii* NGR_c31990 and *E. meliloti* SM11_chr3272)^21^,^23^. It has previously been shown that plants obtaining nitrogen from symbiosis require higher levels of phosphorus for optimal growth than do plants grown with nitrogen fertilizers^25^, and it is tempting to speculate a role in inorganic phosphate-solubilization to enable nodule bioavailability. Another example is CopC domain (PF04234) candidates that showed at least 16X induction in both RNA-seq experiments (*E. fredii* NGR_b06130 and *E. meliloti* SM11_pC0976). In the phytopathogen *Pseudomonas syringae*, CopC has been implicated in mediating copper resistance by binding and sequestering copper in the periplasm^26^ and is believed to function in copper trafficking into cells^27^,^28^. Copper is a cofactor of the high-affinity *cbb3*-type (heme-copper sub-family) cytochrome oxidase, encoded by the *fixNOQP* operon, that terminates the symbiosis-specific respiratory chain of rhizobia^29^,^30^ and CopC may play a role in trafficking copper to *cbb3*-type cytochrome oxidases. In this regard, it is interesting to note that in *E. meliloti* genomes, the locus encoding the CopC domain protein is found downstream of a pSym cluster of *fix* genes including *fixNOQP* and *fixGHISK*. A caveat of employing the EXP criterion is that false negatives are likely because of possible limitations of the method, such as inability to detect significant changes in genes with typically low levels of expression such as regulators.

Within the over-represented subset, we also observed Pfams with primarily eukaryal origin, i. e., the majority of all known sequences that are assigned to these Pfams originated from eukaryal genomes. This led to the hypothesis that RNB may produce eukarya-like factors in order to better interact with, or modulate plant host responses. Indeed, the notion of horizontal gene transfer from a plant host to its bacterial resident has been previously explored^31^,^32^, although merely speculative here. We systematically looked for Pfams of primarily eukaryal origin within the RNB-over-represented set and identified 5 Pfam domains. However, to factor in a previously described observation of shared mechanisms of virulence between plant and animal pathogens, or shared strategies for infection and adaptation to growth within the eukaryal host between pathogens and symbionts^33^,^34^, we extended the search beyond simply “eukaryal origin” to those recruiting sequences from a limited group of prokaryotic lineages (such as primarily Family Rhizobiaceae, or including alpha-proteobacterial pathogens (e.g., *Brucella s*pp. and *Bartonella* spp.). This additional data filtering criterion was designated “LPD” for limited phylogenetic distribution (Fig. 2).

Intriguing examples of these LPD Pfams (Supplementary Table 4) that were over-represented in the RNB included the sterile alpha motif (SAM) domain (PF00536), PAN domain (PF00024), and many DUFs (e.g., PF06191). SAM domains are described as protein interaction modules involved in developmental processes in diverse eukarya. Almost 80% of the total number of sequences assigned to this Pfam within the IMG database belonged to Domain Eukarya. RNB SAM candidates are large multidomain proteins predominantly associated with adenylate guanylate cyclase (PF00211) and AAA ATPase (PF13191) domains, and a role in modulating host response seems likely. The PAN domain (PF00024) has a described role in mediating protein-protein or protein-carbohydrate interactions in eukarya. Again, many RNB PAN domain candidates are large multi-domain sequences associated with alpha-2-macroglobulin domains (PF11974, PF01835, PF07703, PF00207, PF10569) which were themselves not over-represented in the RNB set, however, we speculate the presence of the N-terminal PAN domain (perhaps due to a gene fusion event) may confer a new utility or specificity relevant to RNB activity within its eukaryal host. PAN candidates from eukaryal parasites (*Toxoplasma gondii, Sarcocystis muris*) are characterized as lectin adhesins mediating host binding and invasion by such parasites^35^,^36^. A similar role may be proposed for the RNB PAN domain candidates; this is supported by the presence of a classical signal peptidase I cleavage site (predicted by SignalP) for secretion in most instances.

The third obvious data-filtering criterion was therefore to determine if over-represented Pfam candidates were potentially secreted (see Methods), in order to identify products that may specifically interact with host cell components. Out of the list of 437 over-represented Pfams, 39 had a major proportion (≧50%) of assigned sequences bearing a SignalP motif. For Pfams not meeting this criterion, it is still possible that candidates may be translocated directly into the plant milieu by one of many protein secretion systems (e.g., Type III, Type IV) encoded by the RNB. Only 3 Pfam domains met all three criteria (EXP, LPD, Secretion), while 30 Pfams (out of the over-represented 437) satisfied two or more. Overall, we put forward 184 Pfam domains fulfilling one or more criteria to be designated as determinants of plant interaction or “PID” (Fig. 2, Supplementary Table 4). A proposed role for these can encompass phytostimulation, biocontrol, rhizosphere competence, stress tolerance, in addition to nodulation (including symbiotic signaling, triggering endocytosis and host cell differentiation) and nitrogen fixation. Out of 184 PID Pfams, at least 10 were clearly associated with nodulation and nitrogen fixation, about 8 Pfams may be assigned a regulatory role, and 74 Pfams were implicated in secondary metabolite degradation or synthesis (based on cross-referencing with Pfams used by AntiSMASH^37^ for secondary metabolite operon identification). For example, a role in the hydrolysis of host secondary metabolites is proposed for Epoxide hydrolase N terminus (PF06441) candidates that are mostly predicted to be secreted, however, a role in synthesis may be possible in a few instances where co-localized polyketide biosynthesis genes are present (e.g., *Mesorhizobium loti* USDA 3471). A second example is the berberine-like domain (PF08031), with limited phylogenetic distribution, which typically is found in plant proteins that are involved in the synthesis of isoquinoline alkaloids as a pathogen defense response^38^. Plant berberine bridge enzymes are highly induced during various defense responses, when they may contribute to the oxidative burst leading to cell death, through H_2_O_2_ synthesis; intriguingly, they have been found in the secretome of the phytopathogen *Phytophthora infestans,* where they have been postulated to play a role in virulence^39^. In many RNB strains, the presence of an upstream decarboxylase gene with a putative role in alkaloid synthesis^40^,^41^ favors a role in secondary metabolite biosynthesis, therefore a role in biocontrol of plant pathogens may be conjectured. In instances where domain candidates appear without a co-localized cognate function, a role in the degradation of plant alkaloids may be hypothesized, as seen in *Arthrobacter* spp.^42^ In general, the biological activity of secondary metabolites produced by plant growth promoting bacteria ranges from antimicrobial to phytostimulatory^43^,^44^.

A large number of the 184 PID Pfams were DUFs - DUF2950 (PF11453) and DUF3300 (PF11737) were particularly compelling examples that showed significant induction in both RNA-seq transcriptome studies^21^,^23^, and possessed signal peptides for putative secretion (Supplementary Table 4). Furthermore, the CDS encoding these domains were universally co-localized (DUF3300 is upstream of DUF2950), suggesting cognate function. No other hints regarding their function could be gleaned based on gene neighborhood, and no characterized members were found in public databases.

**Figure 3:**
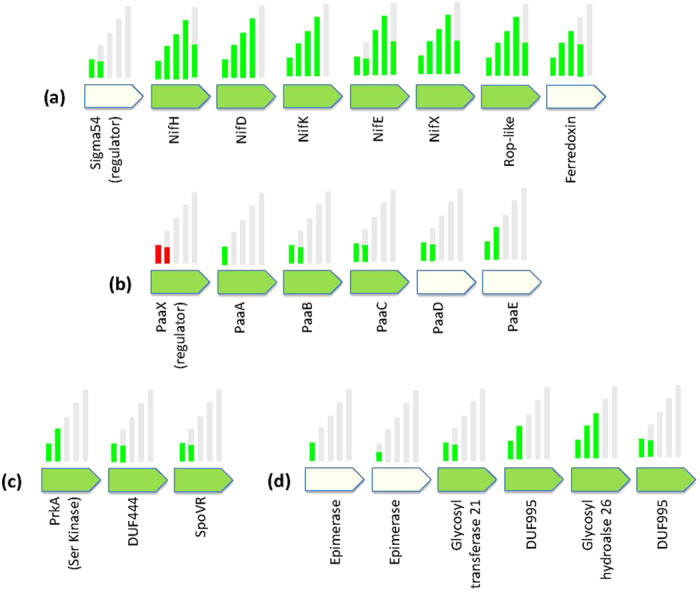
Operons encoding putative determinants of plant interaction. Examples of PID Pfam candidates (colored green) that are co-localized in a putative operon in *E. meliloti* strain 2011. Log_2_ fold increase of transcript in the *E. meliloti* transcriptome experiment^21^ is shown as green bars above the CDS, ranging from 2 to 10. Red bar denotes decrease in transcript abundance in tested condition. *Depicted lengths of coding sequences are not to scale.*

Many PID candidates were also clearly arranged in discrete operons (Fig. 3) – predictably, the nitrogen fixation Pfams were co-localized in a large operon (Fig. 3a). Another notable PID operon was involved in phenyl acetic acid synthesis (Fig. 3b), which is a potential phytohormone (auxinomimetic) with a demonstrated role in nodulation and regulation of nitrogen fixation by *Frankia* spp.^45^, or may function as an antimicrobial agent as suggested for *Azospirillum brasilense*^46^. Other PID operons have more cryptic functions, for example, the operon depicted in Fig. 3c is comprised of a serine protein kinase, a DUF and an unknown gene tenuously associated with sporulation (SpoVR). Certainly bacterial serine kinases have been previously implicated in mediating host-pathogen interactions^47^, and a similar role in mediating plant interaction may be suggested for this operon. Constituents of the operon depicted in Fig. 3d appear to be mostly involved in carbohydrate metabolism and a role in secondary metabolite synthesis is suspected.

In addition to the strategy presented above, we also employed a statistical analysis using Fisher’s Exact Test on pairwise comparisons of gene counts for each Pfam from 163 RNB genomes versus 69 non-phytosphere-associated control or NC genomes described above (see Methods for details). A total of 19 Pfam domains were significantly different in at least 25% of the pairings with a Benjamin-Hochberg P-Value correction cutoff of <0.05 (Supplementary Table 5). Almost all of these significantly “enriched” domains were non-overlapping with the above discussed “over-represented” set because this method teases out domains that contain increased functional potential within the RNB, and likely results from lifestyle-specific expansions of gene families in the RNB compared to the NC genomes. The majority of the domains captured in the enriched set were associated with either transporter or regulatory functions. A role in microbe-host interactions may be postulated for many - for example, a PF13407 (periplasmic binding protein domain) candidate in *E. meliloti* (MocB) is involved in the uptake and degradation of a nodule-specific compound, rhizopine, which plays an important role in symbiosis^48^. Also, many of the candidate genes for this Pfam - 26 out of 38 PF13407 candidates show >2X induction in *Ensifer meliloti* transcriptome experiments^21^. Similarly, PF00211 (adenylate guanylate cyclase domain) candidates show significant induction in transcriptomic studies^21^,^23^ – these genes are involved in the formation of the secondary messengers, cAMP and cGMP^49^, which are triggered by an unknown plant host signal, and involved in establishing infection and symbiosis^50^,^51^,^52^,^53^. Secondary messenger signaling plays a major role in coordinating virulence gene expression in animal pathogens, as well as suppressing eukaryal host immune responses^54^. A list of these relatively-enriched candidate Pfams is presented in Supplementary Table 5.

In conclusion, we have performed an all-inclusive sequencing and analysis of RNB genomes across taxonomic genera, plant host types and biogeographical origins, providing an important scientific resource, and identifying a novel repertoire of determinants of the RNB lifestyle, including those with potential plant beneficial effects. We anticipate that a more comprehensive understanding of these mechanisms will aid the quest to extend N_2_-fixation to non-legume crops, a goal described as essential for future sustainable food production^55^. An additional outcome is a furthering of our appreciation of the role of the RNB in plant growth promotion beyond its known biofertilization effects. Indeed, experimental validation including quantifying the production and relative contribution of these putative effectors of plant growth, relative to the effects of nitrogen fixation, will be very enlightening.

## Methods

### Sequencing, assembly, annotation

The draft genomes of RNB strains were generated at the DOE Joint Genome Institute (JGI) using Illumina technology^56^. For all genomes, we constructed and sequenced an Illumina short-insert paired-end library with an average insert size of 270 bp. All general aspects of library construction and sequencing performed at the JGI can be found at the JGI website (http://www.jgi.doe.gov). The details of sequencing and assembly of individual genomes are reported in the respective genome publications in the Standards in Genome Sciences (http://www.standardsingenomics.org/index.php/sigen/index).

Genomes were annotated by the DOE-JGI genome annotation pipeline^57^. Briefly, protein-coding genes (CDSs) were identified using Prodigal^58^, followed by a round of automated and manual curation using the JGI GenePrimp pipeline^59^. Non-coding genes and miscellaneous features were predicted using tRNAscan-SE^60^, RNAMMer^61^, Rfam^62^. The predicted CDSs were translated and transmembrane regions and signal peptides were predicted using TMHMM^63^, and SignalP^64^. Functional annotation and additional analyses were performed within the Integrated Microbial Genomes (IMG-ER) platform (https://img.jgi.doe.gov/cgi-bin/mer/main.cgi)^65^ including searches against IMG non-redundant database, UniProt, TIGRFam, Pfam, PRIAM, KEGG, COG, and InterPro databases. These data sources were combined to assert a product description for each predicted protein. General genome assembly and annotation statistics are presented in Supplementary Table 2. All available genomic data and annotations may be accessed through the IMG portal (https://img.jgi.doe.gov/cgi-bin/mer/main.cgi).

### Statistical analysis of significantly different or “enriched” Pfams in RNB versus control sets

Pairwise comparisons of gene counts for each Pfam for 163 RNB genomes versus 69 control genomes (163 * 69 = 11,247 comparisons) were performed. Using Fisher’s Exact Test, Pfams that were significantly different in at least 25% of the pairings with a Benjamin-Hochberg P-Value correction cutoff of <0.05 were identified. Normalization of gene counts was determined to be unnecessary since no consistent correlation between number of Pfam hits per genome and genome size were found. Results of this pairwise comparison are presented in Supplementary Table 5.

## Additional Information

**How to cite this article**: Seshadri, R. *et al.* Discovery of Novel Plant Interaction Determinants from the Genomes of 163 Root Nodule Bacteria. *Sci. Rep.* **5**, 16825; doi: 10.1038/srep16825 (2015).

## Supplementary Material

Supplementary Information
