# Supplementary material for: Discovery of Novel Plant Interaction Determinants from the Genomes of 163 Root Nodule Bacteria
Source: Sci Rep. 2015 Nov 20;5:16825. doi: 10.1038/srep16825 (PMC4653623; doi:10.1038/srep16825)
Supplement: Supplementary Information [file srep16825-s1.pdf]

SUPPLEMENTARY TABLES

Discovery of Novel Plant Interaction Determinants from the Genomes of 163 Root Nodule Bacteria

Rekha Seshadri[1], Wayne G. Reeve[2], Julie K. Ardley[2], Kristin Tennessen[1], Tanja Woyke[1], Nikos C. Kyrpides[1] and Natalia N. Ivanova[1]

[1]Department of Energy Joint Genome Institute, Walnut Creek, USA,

[2]Centre for Rhizobium Studies, School of Veterinary and Life Sciences, Murdoch University, Murdoch 6150, Australia

Corresponding author: Rekha Seshadri, email: rseshadri@lbl.gov

Supplementary Table 1. List of RNB strains analyzed in this study

IMG taxon_oid	Genome Name	Genome Reference	Taxonomic Class	Primary Plant Host Name	Isolation Country	Genome Size	Gene Count	GC %	CDS Count	Scaffold Count	Biome
641228476	Azorhizobium caulinodans ORS 571	Dreyfus, B., Garcia, J. L. & Gillis, M. (1988). Characterization of Azorhizobium caulinodans gen. nov., sp. nov., a stem-nodulating nitrogen-fixing bacterium isolated from Sesbania rostrata. International Journal of Systematic Bacteriology 38, 89-98.	Alphaproteobacteria	Sesbania rostrata	Senegal	5369772	4781	0.67	4718	1	Tropical and subtropical grasslands, savannas and shrublands
2513237087	Azorhizobium doebereinerae UFLA1-100	this study	Alphaproteobacteria	Sesbania virgata	Brazil	5817514	5415	0.69	5347	104	Tropical and subtropical moist broadleaf forest
2513237145	Bradyrhizobium elkanii USDA 3254	this study	Alphaproteobacteria	Phaseolus acutifolius	USA	8979722	8564	0.64	8487	85	Deserts and xeric shrublands
2513237096	Bradyrhizobium elkanii USDA 3259	this study	Alphaproteobacteria	Phaseolus lunatus	USA	8722461	8327	0.64	8253	101	Temperate grasslands, savannas and grasslands
2517572143	Bradyrhizobium elkanii USDA 76	this study	Alphaproteobacteria	Glycine max	USA	9484767	9151	0.64	9060	2	Temperate broadleaf and mixed forests
2513237137	Bradyrhizobium elkanii USDA 94	this study	Alphaproteobacteria	Glycine max	USA	9558895	9129	0.64	9057	164	Temperate broadleaf and mixed forests
2513237101	Bradyrhizobium elkanii WSM1741	this study	Alphaproteobacteria	Rhynchosia minima	Australia	7952346	7649	0.62	7581	69	Deserts and xeric shrublands
2513237098	Bradyrhizobium elkanii WSM2783	this study	Alphaproteobacteria	Leobordia carinata	South Africa	9902361	9734	0.62	9641	217	Montane grasslands and shrublands
2516653048	Bradyrhizobium genosp. SA-4 CB756	this study	Alphaproteobacteria	Macrotyloma africanum	Zimbabwe	9826880	9456	0.64	9373	2	Tropical and subtropical grasslands, savannas and shrublands
637000038	Bradyrhizobium japonicum USDA 110	Kaneko T, Nakamura Y, Sato S, Minamisawa K, Uchiumi T, Sasamoto S, Watanabe A, Idesawa K, Iriguchi M, Kawashima K, Kohara M, Matsumoto M, Shimpo S, Tsuruoka H, Wada T, Yamada M, Tabata S. (2002). Complete genomic sequence of nitrogen-fixing symbiotic bacterium Bradyrhizobium japonicum USDA110. DNA Research 9(6):189-97.	Alphaproteobacteria	Glycine max	USA	9105828	8402	0.64	8317	1	Temperate grasslands, savannas and grasslands
2513237095	Bradyrhizobium japonicum USDA 122	this study	Alphaproteobacteria	Glycine max	USA	8976980	8530	0.64	8451	101	Temperate broadleaf and mixed forests
2528768022	Bradyrhizobium japonicum USDA 123	this study	Alphaproteobacteria	Glycine max	USA	1E+07	10573	0.63	10476	517	Temperate grasslands, savannas and grasslands
2517093001	Bradyrhizobium japonicum USDA 124	this study	Alphaproteobacteria	Glycine max	USA	9002274	8621	0.64	8509	123	Temperate grasslands, savannas and grasslands
2513237102	Bradyrhizobium japonicum USDA 135	this study	Alphaproteobacteria	Glycine max	USA	7703324	7923	0.64	7858	547	Temperate grasslands, savannas and grasslands
2513237148	Bradyrhizobium japonicum USDA 38	this study	Alphaproteobacteria	Glycine max	Japan	9608975	9258	0.64	9173	107	Temperate broadleaf and mixed forests
2513237139	Bradyrhizobium japonicum USDA 4	this study	Alphaproteobacteria	Glycine max	USA	8737671	8243	0.64	8169	97	Temperate grasslands, savannas and grasslands
2513237097	Bradyrhizobium japonicum USDA 6	this study	Alphaproteobacteria	Glycine max	Japan	9131141	8736	0.64	8655	87	Temperate broadleaf and mixed forests
2511231207	Bradyrhizobium japonicum USDA 6	Kaneko T, Maita H, Hirakawa H, Uchiike N, Minamisawa K, Watanabe A, Sato S. Complete Genome Sequence of the Soybean Symbiont Bradyrhizobium japonicum Strain USDA6T. (2011). Genes (Basel). 2(4):763-87.	Alphaproteobacteria	Glycine Max	Japan	9207384	8886	0.64	8829	1	Temperate broadleaf and mixed forests
2524023210	Bradyrhizobium sp. Ai1a-2	this study	Alphaproteobacteria	Andira inermis	Costa Rica	9029266	8584	0.63	8482	246	Tropical and subtropical moist broadleaf forest
2508501128	Bradyrhizobium sp. ARR65	this study	Alphaproteobacteria	Stylosanthes viscosa	Australia	8613869	8310	0.62	8246	139	Tropical and subtropical grasslands, savannas and shrublands
640427103	Bradyrhizobium sp. BTAi1	Giraud E, Moulin L, Vallenet D, Barbe V, Cytryn E, et al. (2007) Legumes symbioses: absence of Nod genes in photosynthetic bradyrhizobia. Science 316: 1307–1312.	Alphaproteobacteria	Aeschynomene indica	USA	8493513	7819	0.65	7741	2	Temperate grasslands, savannas and grasslands
2524023205	Bradyrhizobium sp. Cp5.3	this study	Alphaproteobacteria	Centrosema pubescens	Panama	8918781	8545	0.63	8465	148	Tropical and subtropical moist broadleaf forest
2513237104	Bradyrhizobium sp. EC3.3	this study	Alphaproteobacteria	Erythrina costaricensis	Panama	1E+07	9729	0.63	9656	154	Tropical and subtropical moist broadleaf forest
2519103088	Bradyrhizobium sp. ORS 375	Mornico, D., Miché, L., Béna, G. & other authors (2011). Comparative genomics of Aeschynomene symbionts: insights into the ecological lifestyle of Nod-Independent photosynthetic bradyrhizobia. Genes 3, 35-61	Alphaproteobacteria	Aeschynomene sp.	Senegal	7859468	7143	0.65	7078	497	Tropical and subtropical grasslands, savannas and shrublands
640427104	Bradyrhizobium sp. ORS278	Giraud E, Moulin L, Vallenet D, Barbe V, Cytryn E, et al. (2007) Legumes symbioses: absence of Nod genes in photosynthetic bradyrhizobia. Science 316: 1307–1312.	Alphaproteobacteria	Aeschynomene sensitiva	Senegal	7456587	6825	0.66	6752	1	Tropical and subtropical grasslands, savannas and shrublands
2513237268	Bradyrhizobium sp. ORS285	Adeline Renier, Fabienne Maillet, Joel Fardoux, Véréna Poinsot, Eric Giraud, and Nico Nouwen. Photosynthetic Bradyrhizobium Sp. Strain ORS285 Synthesizes 2-O-Methylfucosylated Lipochitooligosaccharides for nod Gene–Dependent Interaction with Aeschynomene Plants. (2012). Molecular Plant Microbe Interactions. Volume 24, Number 12.	Alphaproteobacteria	Aeschynomene afraspera	Senegal	7602254	6842	0.65	6778	301	Tropical and subtropical grasslands, savannas and shrublands
2513237312	Bradyrhizobium sp. STM 3809	Damien Mornico, Lucie Miché, Gilles Béna, Nico Nouwen, André Verméglio, David Vallenet, Alexander A.T. Smith, Eric Giraud, Claudine Médigue and Lionel Moulin. Comparative Genomics of Aeschynomene Symbionts: Insights into the Ecological Lifestyle of Nod-Independent Photosynthetic Bradyrhizobia. (2012). Genes 3, 35-61.	Alphaproteobacteria	Aeschynomene sp.	French Guiana	7311748	6699	0.66	6637	803	Tropical and subtropical moist broadleaf forest
2524023228	Bradyrhizobium sp. th.b2	this study	Alphaproteobacteria	Amphicarpaea bracteata	USA	1E+07	9917	0.63	9809	266	Temperate broadleaf and mixed forests
2513237141	Bradyrhizobium sp. TV2a.2	this study	Alphaproteobacteria	Tachigali versicolor	Panama	8496279	8181	0.62	8109	87	Tropical and subtropical moist broadleaf forest
2513237136	Bradyrhizobium sp. USDA 3384	this study	Alphaproteobacteria	Crotalaria paulina	Brazil	9800269	9313	0.64	9240	51	Tropical and subtropical grasslands, savannas and shrublands
2508501042	Bradyrhizobium sp. WSM1253	this study	Alphaproteobacteria	Ornithopus compressus	Greece	8719808	8498	0.63	8432	2	Mediterranean forests, woodlands and scrub
2507262055	Bradyrhizobium sp. WSM1417	this study	Alphaproteobacteria	Lupinus sp.	Chile	8048963	7772	0.63	7695	1	Mediterranean forests, woodlands and scrub
2513237092	Bradyrhizobium sp. WSM1743	this study	Alphaproteobacteria	Indigofera sp.	Australia	8341956	7983	0.63	7908	163	Deserts and xeric shrublands
2513237153	Bradyrhizobium sp. WSM2254	this study	Alphaproteobacteria	Acacia sp.	Australia	9086516	8597	0.64	8528	100	Temperate broadleaf and mixed forests
2513237161	Bradyrhizobium sp. WSM2793	this study	Alphaproteobacteria	Rhynchosia totta	South Africa	8871253	8414	0.64	8349	84	Montane grasslands and shrublands
2513237094	Bradyrhizobium sp. WSM3983	this study	Alphaproteobacteria	Kennedia coccinea	Australia	8789602	8469	0.63	8402	70	Deserts and xeric shrublands
2515154112	Bradyrhizobium sp. WSM4349	this study	Alphaproteobacteria	Syrmatium glabrum	USA	8294334	8114	0.63	8033	104	Mediterranean forests, woodlands and scrub
2508501009	Bradyrhizobium sp. WSM471	this study	Alphaproteobacteria	Ornithopus pinnatus	Australia	7784016	7430	0.63	7372	1	Mediterranean forests, woodlands and scrub
2508501124	Burkholderia dilworthii WSM3556	this study	Betaproteobacteria	Lebeckia ambigua	South Africa	7679067	7123	0.62	7059	140	Mediterranean forests, woodlands and scrub
2513237083	Burkholderia mimosarum LMG 23256	this study	Betaproteobacteria	Mimosa pigra	Taiwan	8410967	7886	0.64	7801	268	Tropical and subtropical moist broadleaf forest
2513237082	Burkholderia mimosarum STM 3621	this study	Betaproteobacteria	Mimosa pudica	French Guiana	8640282	8145	0.64	8062	268	Tropical and subtropical moist broadleaf forest
2515154189	Burkholderia nodosa DSM 21604	unpublished	Betaproteobacteria	Mimosa scabrella	Brazil	9627966	8862	0.64	8777	114	Deserts and xeric shrublands

2518645580	Burkholderia phenoliruptrix BR3459	de Oliveira Cunha C, Goda Zuleta LF, Paula de Almeida LG, Prioli Ciapina L, Lustrino Borges W, Pitard RM, Baldani JI, Straliotto R, de Faria SM, Hungria M, Sousa Cavada B, Mercante FM, Ribeiro de Vasconcelos AT. Complete genome sequence of Burkholderia phenoliruptrix BR3459a (CLA1), a heat-tolerant, nitrogen-fixing symbiont of Mimosa flocculosa. (2012). J Bacteriol 194(23):6675-6	Betaproteobacteria	Mimosa flocculosa	Brazil	7651131	6605	0.63	6525	3	Deserts and xeric shrublands
642555112	Burkholderia phymatum STM 815	Moulin L, Klonowska A, Caroline B, Booth K, Vriezen JA, Melkonian R, James EK, Young JP, Bena G, Hauser L, Land M, Kyrpides N, Bruce D, Chain P, Copeland A, Pitluck S, Woyke T, Lizotte-Waniewski M, Bristow J, Riley M. Complete Genome sequence of Burkholderia phymatum STM815(T), a broad host range and efficient nitrogen-fixing symbiont of Mimosa species. (2014). Stand Genomic Sci. 9(3):763-74.	Betaproteobacteria	Mimosa sp.	French Guiana	8676562	7574	0.62	7496	4	Tropical and subtropical moist broadleaf forest
646564515	Burkholderia sp. CCGE1002	Ormeño-Orrillo E, Rogel MA, Chueire LM, Tiedje JM, Martínez-Romero E, Hungria M. Genome sequences of Burkholderia sp. strains CCGE1002 and H160, isolated from legume nodules in Mexico and Brazil. (2012), J Bacteriol. 194(24):6927.	Betaproteobacteria	Mimosa occidentalis	Mexico	7884858	7358	0.63	7261	4	Tropical and subtropical dry broadleaf forest
2515154122	Burkholderia sp. JPY251	this study	Betaproteobacteria	Mimosa velloziana	Brazil	8609520	7983	0.63	7893	122	Deserts and xeric shrublands
2526164713	Burkholderia sp. JPY366	this study	Betaproteobacteria	Mimosa misera	Brazil	6780608	6146	0.64	6070	69	Deserts and xeric shrublands
2513237166	Burkholderia sp. UYPR1.413	this study	Betaproteobacteria	Parapiptadenia rigida (Angico)	Uruguay	1E+07	9836	0.62	9759	336	Tropical and subtropical grasslands, savannas and shrublands
2510065045	Burkholderia sprentiae WSM5005	this study	Betaproteobacteria	Lebeckia ambigua	South Africa	7761063	7223	0.63	7147	8	Mediterranean forests, woodlands and scrub
2512047030	Burkholderia tuberum STM 678	Angus AA, Agapakis CM, Fong S, Yerrapragada S, Estrada-de los Santos P, Yang P, Song N, Kano S, Caballero-Mellado J, de Faria SM, Dakora FD, Weinstock G, Hirsch AM. Plant-associated symbiotic Burkholderia species lack hallmark strategies required in mammalian pathogenesis. (2014). PLoS One. 9(1):e83779.	Betaproteobacteria	Cyclopia sp.	South Africa	9031815	8658	0.63	8534	284	Mediterranean forests, woodlands and scrub
2516653074	Burkholderia tuberum WSM4176	this study	Betaproteobacteria	Lebeckia ambigua	South Africa	9065247	8497	0.63	8369	13	Mediterranean forests, woodlands and scrub
2524023212	Cupriavidus sp. AMP6	this study	Betaproteobacteria	Mimosa asperata	USA	7579563	7130	0.65	7033	260	Deserts and xeric shrublands
2513237163	Cupriavidus sp. UYPR2.512	this study	Betaproteobacteria	Parapiptadenia rigida	Uruguay	7858949	7487	0.65	7411	365	Tropical and subtropical grasslands, savannas and shrublands
644736347	Cupriavidus taiwanensis LMG 19424	Amadou C, Pascal G, Mangenot S, Glew M, Bontemps C, Capela D, Carrère S, Cruveiller S, Dossat C, Lajus A, Marchetti M, Poinsot V, Rouy Z, Servin B, Saad M, Schenowitz C, Barbe V, Batut J, Médigue C, Masson-Boivin C. Genome sequence of the beta-rhizobium Cupriavidus taiwanensis and comparative genomics of rhizobia. (2008). Genome Res. 18(9):1472-83	Betaproteobacteria	Mimosa pudica	Taiwan	6476522	5986	0.67	5897	3	Tropical and subtropical moist broadleaf forest
2513237150	Cupriavidus taiwanensis STM 6018	this study	Betaproteobacteria	Mimosa pudica	French Guiana	6553639	5925	0.67	5864	80	Tropical and subtropical moist broadleaf forest
2513237165	Cupriavidus taiwanensis STM 6070	this study	Betaproteobacteria	Mimosa pudica	New Caledonia	6771773	6182	0.67	6118	107	Tropical and subtropical moist broadleaf forest
2512047086	Ensifer arboris LMG 14919	this study	Alphaproteobacteria	Prosopis chilensis	Sudan	6850303	6545	0.62	6461	7	Tropical and subtropical grasslands, savannas and shrublands
2547132261	Ensifer fredii GR64	Torres Tejerizo G, Lozano L, González V, Bustos P, Romero D, Brom S. Draft genome sequence of the bean-nodulating Sinorhizobium fredii strain GR64. (2012). J Bacteriol. 194(24):6978.	Alphaproteobacteria	Phaseolus vulgaris	Spain	6959392	6630	0.62	6551	216	Mediterranean forests, woodlands and scrub
2517572023	Ensifer fredii HH103	Weidner S, Becker A, Bonilla I, Jaenicke S, Lloret J, Margaret I, Pühler A, Ruiz-Sainz JE, Schneiker-Bekel S, Szczepanowski R, Vinardell JM, Zehner S, Göttfert M. Genome sequence of the soybean symbiont Sinorhizobium fredii HH103. (2012). J Bacteriol. 2012 194(6):1617-8.	Alphaproteobacteria	Glycine max	China	7221188	6851	0.62	6789	10	Tropical and subtropical moist broadleaf forest
643692032	Ensifer fredii NGR234	Schmeisser, C., Liesegang, H., Krysciak, D. & other authors (2009). Rhizobium sp strain NGR234 possesses a remarkable number of secretion systems. Applied and Environmental Microbiology 75, 4035-4045	Alphaproteobacteria	Lablab purpureus	Papua New Guine	6891900	6437	0.62	6376	3	Tropical and subtropical moist broadleaf forest
2514885035	Ensifer fredii USDA 257	Schuldes J, Rodriguez Orbegoso M, Schmeisser C, Krishnan HB, Daniel R, Streit WR. Complete genome sequence of the broad-host-range strain Sinorhizobium fredii USDA257. (2012), J Bacteriol. 194(16):4483.	Alphaproteobacteria	Glycine soja	China	7032323	6855	0.62	6793	20	Temperate broadleaf and mixed forests
2513237089	Ensifer medicae Di28	this study	Alphaproteobacteria	Medicago arabica	Sardinia	6553624	6469	0.61	6394	104	Mediterranean forests, woodlands and scrub
2512875026	Ensifer medicae WSM1115	this study	Alphaproteobacteria	Medicago polymorpha	Greece	6861065	6872	0.61	6789	7	Mediterranean forests, woodlands and scrub
2513237156	Ensifer medicae WSM1369	this study	Alphaproteobacteria	Medicago sphaerocarpos	Sardinia	6402557	6735	0.61	6656	307	Mediterranean forests, woodlands and scrub
2513237160	Ensifer medicae WSM244	this study	Alphaproteobacteria	Medicago polymorpha	Iraq	6650282	6495	0.61	6427	91	Temperate grasslands, savannas and grasslands
640753051	Ensifer medicae WSM419	this study	Alphaproteobacteria	Medicago murex	Sardinia	6817576	6599	0.61	6518	4	Mediterranean forests, woodlands and scrub
2517487022	Ensifer medicae WSM4191	this study	Alphaproteobacteria	Melilotus siculus	Australia	7227575	7099	0.61	7010	6	Mediterranean forests, woodlands and scrub
637000269	Ensifer meliloti 1021	Galibert, F., Finan, T. M., Long, S. R. & other authors (2001). The composite genome of the legume symbiont Sinorhizobium meliloti. Science 293, 668-672	Alphaproteobacteria	Medicago sativa	Australia	6691694	6295	0.62	6212	3	Temperate broadleaf and mixed forests
2551306089	Ensifer meliloti 1A42	unpublished	Alphaproteobacteria	Medicago sativa	Iran	7162724	7454	0.62	7360	315	Deserts and xeric shrublands
2562617130	Ensifer meliloti 2011	Sallet E, Roux B, Sauviac L, Jardinaud MF, Carrère S, Faraut T, de Carvalho-Niebel F, Gouzy J, Gamas P, Capela D, Bruand C (2013) Next-generation annotation of prokaryotic genomes with EuGene-P: application to Sinorhizobium meliloti 2011 DNA Res. 20(4):339-54	Alphaproteobacteria	Medicago sativa	Australia	6693185	8251	0.62	6312	3	Temperate broadleaf and mixed forests
2515154107	Ensifer meliloti 4H41	this study	Alphaproteobacteria	Phaseolus vulgaris	Tunisia	6795637	6422	0.62	6350	47	Deserts and xeric shrublands
2551306084	Ensifer meliloti 5A14	unpublished	Alphaproteobacteria	Medicago sativa	Iran	8942552	8870	0.62	8728	217	Deserts and xeric shrublands
2551306090	Ensifer meliloti A0641M	unpublished	Alphaproteobacteria	Medicago sativa	Italy	7953713	8520	0.62	8412	451	Mediterranean forests, woodlands and scrub

2551306087	Ensifer meliloti A0643DD	unpublished	Alphaproteobacteria	Medicago sativa	Italy	7351905	7849	0.62	7755	678	Mediterranean forests, woodlands and scrub
2551306085	Ensifer meliloti AE608H	unpublished	Alphaproteobacteria	Medicago sativa	Italy	7347181	7285	0.62	7181	223	Mediterranean forests, woodlands and scrub
2551306086	Ensifer meliloti AK11	unpublished	Alphaproteobacteria	Medicago falcata	Kazakhstan	6843938	6988	0.62	6902	424	Temperate grasslands, savannas and grasslands
2511231052	Ensifer meliloti AK58	Galardini M, Bazzicalupo M, Biondi E, Brambilla E, Brilli M, Bruce D, Chain P, Chen A, Daligault H, Davenport KW, Deshpande S, Detter JC, Goodwin LA, Han C, Han J, Huntemann M, Ivanova N, Klenk HP, Kyrpides NC, Markowitz V, Mavrommatis K, Mocali S, Nolan M, Pagani I, Pati A, Pini F, Pitluck S, Spini G, Szeto E, Teshima H, Woyke T, Mengoni A. Permanent draft genome sequences of the symbiotic nitrogen fixing Ensifer meliloti strains BO21CC and AK58. (2013). Stand Genomic Sci. 15;9(2):325-33.	Alphaproteobacteria	Medicago falcata	Kazakhstan	6974333	7013	0.62	6934	9	Temperate grasslands, savannas and grasslands
2551306092	Ensifer meliloti AK75	unpublished	Alphaproteobacteria	Medicago lupulina	Kazakhstan	6992595	7636	0.62	7550	700	Temperate grasslands, savannas and grasslands
650716086	Ensifer meliloti AK83	Galardini et al. (2011) Exploring the symbiotic pangenome of the nitrogen-fixing bacterium Sinorhizobium meliloti BMC Genomics, 12:235	Alphaproteobacteria	Medicago falcata	Kazakhstan	7140471	7022	0.62	6954	5	Temperate grasslands, savannas and grasslands
648276728	Ensifer meliloti BL225C	Galardini et al. (2011) Exploring the symbiotic pangenome of the nitrogen-fixing bacterium Sinorhizobium meliloti BMC Genomics, 12:237	Alphaproteobacteria	Medicago sativa	Italy	6968865	6769	0.62	6712	158	Mediterranean forests, woodlands and scrub
2516653046	Ensifer meliloti BO21CC	Galardini M, Bazzicalupo M, Biondi E, Brambilla E, Brilli M, Bruce D, Chain P, Chen A, Daligault H, Davenport KW, Deshpande S, Detter JC, Goodwin LA, Han C, Han J, Huntemann M, Ivanova N, Klenk HP, Kyrpides NC, Markowitz V, Mavrommatis K, Mocali S, Nolan M, Pagani I, Pati A, Pini F, Pitluck S, Spini G, Szeto E, Teshima H, Woyke T, Mengoni A. Permanent draft genome sequences of the symbiotic nitrogen fixing Ensifer meliloti strains BO21CC and AK58. (2013). Stand Genomic Sci. 15;9(2):325-33.	Alphaproteobacteria	Medicago sativa	Italy	6989037	6907	0.62	6805	3	Mediterranean forests, woodlands and scrub
2551306091	Ensifer meliloti C0431A	unpublished	Alphaproteobacteria	Medicago sativa	Italy	7086830	7449	0.62	7373	401	Mediterranean forests, woodlands and scrub
2551306093	Ensifer meliloti C0438LL	unpublished	Alphaproteobacteria	Medicago sativa	Italy	7064773	7332	0.62	7237	492	Mediterranean forests, woodlands and scrub
2519103084	Ensifer meliloti CCNWSX0020	Li Z, Ma Z, Hao X, Wei G. Draft genome sequence of Sinorhizobium meliloti CCNWSX0020, a nitrogen-fixing symbiont with copper tolerance capability isolated from lead-zinc mine tailings. (2012). J Bacteriol. 194(5):1267-8.	Alphaproteobacteria	Medicago lupulina	China	7001588	6900	0.62	6847	233	
2510065056	Ensifer meliloti CIAM1775	this study	Alphaproteobacteria	Medicago lupulina	Kazakhstan	6712855	6516	0.62	6440	108	Temperate grasslands, savannas and grasslands
2523533632	Ensifer meliloti GR4	Martínez-Abarca F, Martínez-Rodríguez L, López-Contreras JA, Jiménez-Zurdo JI, Toro N. Complete Genome Sequence of the Alfalfa Symbiont Sinorhizobium/Ensifer meliloti Strain GR4. (2013). Genome Announc. 1(1). pii: e00174-12.	Alphaproteobacteria	Medicago sativa	Spain	7139558	6826	0.62	6707	5	Mediterranean forests, woodlands and scrub
2513237140	Ensifer meliloti GVPV12	this study	Alphaproteobacteria	Phaseolus vulgaris	Canary Islands	7076289	6806	0.62	6727	106	Mediterranean forests, woodlands and scrub
2551306148	Ensifer meliloti H1	unpublished	Alphaproteobacteria	Medicago sativa	Italy	6923151	7084	0.62	6990	397	Mediterranean forests, woodlands and scrub
2513237143	Ensifer meliloti Mlalz-1	this study	Alphaproteobacteria	Medicago laciniata	Canary Islands	6664116	6388	0.62	6314	99	Mediterranean forests, woodlands and scrub
2513237086	Ensifer meliloti MVII-I	this study	Alphaproteobacteria	Medicago sativa	Germany	7265287	7213	0.62	7133	220	Temperate broadleaf and mixed forests
2513237091	Ensifer meliloti RRI128	this study	Alphaproteobacteria	Medicago truncatula	Australia	6900273	6770	0.62	6683	156	Mediterranean forests, woodlands and scrub
651053067	Ensifer meliloti SM11	Schneiker-Bekel S, Wibberg D, Bekel T, Blom J, Linke B, Neuweger H, Stiens M, Vorhölter FJ, Weidner S, Goesmann A, Pühler A, Schlüter A. The complete genome sequence of the dominant Sinorhizobium meliloti field isolate SM11 extends the S. meliloti pan-genome. (2011). J Biotechnol. 155(1):20-33.	Alphaproteobacteria	Medicago sativa	Germany	7173736	7156	0.62	7093	3	Temperate coniferous forests
2510065057	Ensifer meliloti WSM1022	this study	Alphaproteobacteria	Medicago orbicularis	Greece	6649661	6398	0.62	6323	121	Mediterranean forests, woodlands and scrub
2516143018	Ensifer sp. BR816	this study	Alphaproteobacteria	Leucaena leucocephala	Brazil	6951533	6643	0.62	6554	3	Tropical and subtropical moist broadleaf forest
2509276019	Ensifer sp. TW10	this study	Alphaproteobacteria	Tephrosia wallichii	India	6802256	6546	0.62	6473	57	Deserts and xeric shrublands
2524023207	Ensifer sp. USDA 6670, CC2017	this study	Alphaproteobacteria	Medicago sativa	Australia	6813453	6547	0.62	6465	73	Temperate grasslands, savannas and grasslands
2508501122	Ensifer sp. WSM1721	this study	Alphaproteobacteria	Indigofera sp.	Australia	6292184	6011	0.62	5935	68	Deserts and xeric shrublands
2513237351	Mesorhizobium alhagi CCNWXJ12-2	Zhou, M., Chen, W., Chen, H. & Wei, G. (2012). Draft genome sequence of Mesorhizobium alhagi CCNWXJ12-2T, a novel salt-resistant species isolated from the desert of Northwestern China. Journal of Bacteriology 194, 1261-1262	Alphaproteobacteria	Alhagi sparsifolia	China	6968952	7244	0.63	7195	375	Deserts and xeric shrublands
2513237305	Mesorhizobium amorphae CCNWGS0123	Hao X, Lin Y, Johnstone L, Baltrus DA, Miller SJ, Wei G, Rensing C. Draft genome sequence of plant growth-promoting rhizobium Mesorhizobium amorphae, isolated from zinc-lead mine tailings. (2012). J Bacteriol. 194(3):736-7.	Alphaproteobacteria	Robinia pseudoacacia	China	7293571	7136	0.63	7084	274	Temperate broadleaf and mixed forests
2509276022	Mesorhizobium australicum WSM2073	this study	Alphaproteobacteria	Biserrula pelecinus	Australia	6200534	6080	0.63	6013	1	Mediterranean forests, woodlands and scrub
2509276018	Mesorhizobium ciceri CMG6	this study	Alphaproteobacteria	Cicer arietinum	Tunisia	6910194	6718	0.62	6648	90	Mediterranean forests, woodlands and scrub
649633066	Mesorhizobium ciceri WSM1271	this study	Alphaproteobacteria	Biserrula pelecinus	Sardinia	6690028	6531	0.63	6470	2	Mediterranean forests, woodlands and scrub
2510065059	Mesorhizobium ciceri WSM4083	this study	Alphaproteobacteria	Bituminaria bitumosa	Canary Islands	6847884	6662	0.63	6596	42	Mediterranean forests, woodlands and scrub
2513237164	Mesorhizobium loti CJ3sym	this study	Alphaproteobacteria	Lotus corniculatus	New Zealand	7563725	7401	0.62	7331	70	Temperate grasslands, savannas and grasslands
637000159	Mesorhizobium loti MAFF303099	Kaneko T, Nakamura Y, Sato S, Asamizu E, Kato T, Sasamoto S, Watanabe A, Idesawa K, Ishikawa A, Kawashima K, Kimura T, Kishida Y, Kiyokawa C, Kohara M, Matsumoto M, Matsuno A, Mochizuki Y, Nakayama S, Nakazaki N, Shimpo S, Sugimoto M, Takeuchi C, Yamada M, Tabata S. Complete genome structure of the nitrogen-fixing symbiotic bacterium Mesorhizobium loti. (2000). DNA Res. 7(6):331-8.	Alphaproteobacteria	Lotus corniculatus	Japan	7596297	7356	0.63	7272	3	Temperate broadleaf and mixed forests
2517572076	Mesorhizobium loti NZP2037	this study	Alphaproteobacteria	Lotus divaricatus	New Zealand	7462792	7388	0.63	7318	1	Temperate broadleaf and mixed forests

2512875016	Mesorhizobium loti R7A	this study	Alphaproteobacteria	Lotus corniculatus	New Zealand	6529530	6398	0.63	6323	1	Temperate broadleaf and mixed forests
2512875024	Mesorhizobium loti R88b	this study	Alphaproteobacteria	Lotus corniculatus	New Zealand	7195110	7016	0.62	6950	1	Temperate broadleaf and mixed forests
2513237147	Mesorhizobium loti USDA 3471	this study	Alphaproteobacteria	Lotus corniculatus	New Zealand	7023073	6785	0.63	6714	50	Temperate broadleaf and mixed forests
2503198000	Mesorhizobium opportunistum WSM2075	this study	Alphaproteobacteria	Biserrula pelecinus	Australia	6884444	6747	0.63	6685	1	Mediterranean forests, woodlands and scrub
2508501126	Mesorhizobium sp. WSM1293	this study	Alphaproteobacteria	Lotus ornithopodioides	Greece	6938611	6711	0.62	6640	51	Mediterranean forests, woodlands and scrub
2508501127	Mesorhizobium sp. WSM2561	this study	Alphaproteobacteria	Lessertia diffusa	South Africa	7037543	6910	0.62	6834	111	Mediterranean forests, woodlands and scrub
2513237090	Mesorhizobium sp. WSM3224	this study	Alphaproteobacteria	Otholobium candicans	South Africa	7096802	6951	0.63	6880	72	Mediterranean forests, woodlands and scrub
2508501123	Mesorhizobium sp. WSM3626	this study	Alphaproteobacteria	Lessertia diffusa	South Africa	6283661	6243	0.63	6172	106	Deserts and xeric shrublands
643348564	Methylobacterium nodulans ORS 2060	Marx, C. J., Bringel, F., Chistoserdova, L. et. Al. (2012). Complete genome sequences of six strains of the genus Methylobacterium. Journal of Bacteriology 194, 4746-4748	Alphaproteobacteria	Crotalaria podocarpa	Senegal	8839022	8885	0.68	8791	8	Tropical and subtropical grasslands, savannas and shrublands
641522639	Methylobacterium sp. 4-46	Marx, C. J., Bringel, F., Chistoserdova, L. et. Al. (2012). Complete genome sequences of six strains of the genus Methylobacterium. Journal of Bacteriology 194, 4746-4748	Alphaproteobacteria	Listia bainesii	South Africa	7737025	7125	0.72	7043	3	
2545555834	Methylobacterium sp. WSM2598	this study	Alphaproteobacteria	Listia bainesii	South Africa	8130841	7667	0.71	7551	85	Montane grasslands and shrublands
2508501114	Microvirga lotononidis WSM3557	this study	Alphaproteobacteria	Listia angolensis	Zambia	7082538	7040	0.63	6956	18	Flooded grasslands and savannas
2508501050	Microvirga lupini Lut6	this study	Alphaproteobacteria	Lupinus texensis	USA	9633614	10951	0.62	10864	160	Temperate grasslands, savannas and grasslands
640427137	Rhizobium etli CFN 42, DSM 11541	González, V., Santamaría, R. I., Bustos, P. & other authors (2006). The partitioned Rhizobium etli genome: Genetic and metabolic redundancy in seven interacting replicons. Proceedings of the National Academy of Sciences 103, 3834-3839	Alphaproteobacteria	Phaseolus vulgaris	Mexico	6530228	6117	0.61	6038	7	Tropical and subtropical dry broadleaf forest
642555152	Rhizobium etli CIAT 652	unpublished	Alphaproteobacteria	Phaseolus vulgaris	Colombia	6448048	6132	0.61	6072	4	Tropical and subtropical dry broadleaf forest
2529292711	Rhizobium etli CNPAF512	Maarten Fauvart, Aminael Sánchez-Rodríguez, Serge Beullens, Kathleen Marchal, and Jan Michiels. Genome Sequence of Rhizobium etli CNPAF512, a Nitrogen-Fixing Symbiont Isolated from Bean Root Nodules in Brazil. (2011). J Bacteriol. 193(12): 3158–3159.	Alphaproteobacteria	Phaseolus vulgaris	Brazil	6726123	7586	0.61	7586	402	
2515154134	Rhizobium gallicum bv. gallicum R602sp	this study	Alphaproteobacteria	Phaseolus vulgaris	France	7220242	6977	0.61	6900	38	Temperate broadleaf and mixed forests
2513237159	Rhizobium giardinii bv. giardinii H152	this study	Alphaproteobacteria	Phaseolus vulgaris	France	6810126	6691	0.61	6618	190	Temperate broadleaf and mixed forests
2534681796	Rhizobium grahamii CCGE 502	Althabegoiti MJ, Lozano L, Torres-Tejerizo G, Ormeño-Orrillo E, Rogel MA, González V, Martínez-Romero E. Genome sequence of Rhizobium grahamii CCGE502, a broad-host-range symbiont with low nodulation competitiveness in Phaseolus vulgaris. (2012). J Bacteriol. 194(23):6651-2.	Alphaproteobacteria	Dalea leporina	Mexico	7146037	6898	0.59	6844	80	
2513237093	Rhizobium leguminosarum bv phaseoli FA23	this study	Alphaproteobacteria	Phaseolus vulgaris	Poland	7545552	7285	0.61	7203	66	Temperate broadleaf and mixed forests
2509276052	Rhizobium leguminosarum bv trifolii CC278f	this study	Alphaproteobacteria	Trifolium nanum	USA	8729608	8665	0.61	8582	7	Temperate grasslands, savannas and grasslands
2517287029	Rhizobium leguminosarum bv trifolii SRDI565	this study	Alphaproteobacteria	Trifolium subterraneum	Australia	6905599	6836	0.61	6750	7	Temperate grasslands, savannas and grasslands
2510461076	Rhizobium leguminosarum bv trifolii TA1	this study	Alphaproteobacteria	Trifolium subterraneum	Australia	8618824	8576	0.61	8493	6	Temperate broadleaf and mixed forests
2510065019	Rhizobium leguminosarum bv trifolii WSM16	this study	Alphaproteobacteria	Trifolium uniflorum	Greece	6903379	6798	0.61	6709	6	Mediterranean forests, woodlands and scrub
2508501100	Rhizobium leguminosarum bv trifolii WSM22	this study	Alphaproteobacteria	Trifolium africanum	South Africa	7999455	7827	0.61	7746	4	Montane grasslands and shrublands
2515154115	Rhizobium leguminosarum bv viciae 128C53	this study	Alphaproteobacteria	Pisum sativum	United Kingdom	7609235	7396	0.61	7322	72	Temperate broadleaf and mixed forests
2513237162	Rhizobium leguminosarum bv viciae GB30	this study	Alphaproteobacteria	Pisum sativum	Poland	7468464	7302	0.61	7227	78	Temperate broadleaf and mixed forests
2513237084	Rhizobium leguminosarum bv viciae UPM113	this study	Alphaproteobacteria	Pisum sativum	Italy	7231967	6951	0.61	6870	41	Temperate and subtropical coniferous forest
2513237085	Rhizobium leguminosarum bv viciae UPM113	this study	Alphaproteobacteria	Pisum sativum	Italy	7695351	7462	0.61	7390	49	Temperate broadleaf and mixed forests
2513237103	Rhizobium leguminosarum bv viciae VF39	this study	Alphaproteobacteria	Vicia faba	Germany	7647401	7469	0.61	7392	89	Temperate broadleaf and mixed forests
2509276044	Rhizobium leguminosarum bv viciae WSM14	this study	Alphaproteobacteria	Vicia faba	Greece	7557514	7494	0.61	7418	3	Mediterranean forests, woodlands and scrub
2516653077	Rhizobium leguminosarum bv viciae WSM14	this study	Alphaproteobacteria	Vicia faba	Greece	7555578	7464	0.61	7374	6	Mediterranean forests, woodlands and scrub
2516653085	Rhizobium leguminosarum bv. phaseoli 4292	this study	Alphaproteobacteria	Phaseolus vulgaris	United Kingdom	7346596	7193	0.61	7109	5	Temperate broadleaf and mixed forests
2510065076	Rhizobium leguminosarum bv. trifolii CB782	this study	Alphaproteobacteria	Trifolium semipilosum	Kenya	6703653	6559	0.61	6472	4	Montane grasslands and shrublands
2507525018	Rhizobium leguminosarum bv. trifolii CC283b	this study	Alphaproteobacteria	Trifolium ambiguum	Caucasus	7923462	7731	0.61	7654	11	Temperate grasslands, savannas and grasslands
2517093000	Rhizobium leguminosarum bv. trifolii SRDI94	this study	Alphaproteobacteria	Trifolium subterraneum	Australia	7412387	7406	0.61	7317	5	Temperate grasslands, savannas and grasslands
644736401	Rhizobium leguminosarum bv. trifolii WSM13	this study	Alphaproteobacteria	Trifolium sp.	Greece	7418122	7292	0.61	7232	6	Mediterranean forests, woodlands and scrub
2509276033	Rhizobium leguminosarum bv. trifolii WSM20	this study	Alphaproteobacteria	Trifolium rueppellianum	Ethiopia	7180565	7166	0.61	7080	6	Montane grasslands and shrublands
643348569	Rhizobium leguminosarum bv. trifolii WSM23	this study	Alphaproteobacteria	Trifolium polymorphum	Uruguay	6872702	6643	0.61	6581	5	Temperate grasslands, savannas and grasslands
2509276021	Rhizobium leguminosarum bv. trifolii WSM59	this study	Alphaproteobacteria	Trifolium pallidum	Uruguay	7634384	7481	0.61	7394	2	Temperate grasslands, savannas and grasslands
2515075009	Rhizobium leguminosarum bv. viciae 248	this study	Alphaproteobacteria	Vicia faba	United Kingdom	7288508	7179	0.61	7090	7	Temperate broadleaf and mixed forests
639633055	Rhizobium leguminosarum bv. viciae 3841	Young JP, Crossman LC, Johnston AW, Thomson NR, Ghazoui ZF, Hull KH, Wexler M, Curson AR, Todd JD, Poole PS, Mauchline TH, East AK, Quail MA, Churcher C, Arrowsmith C, Cherevach I, Chillingworth T, Clarke K, Cronin A, Davis P, Fraser A, Hance Z, Hauser H, Jagels K, Moule S, Mungall K, Norbertczak H, Rabbinowitsch E, Sanders M, Simmonds M, Whitehead S, Parkhill J. The genome of Rhizobium leguminosarum has recognizable core and accessory components. (2006). Genome Biol. 7(4):R34.	Alphaproteobacteria	Pisum sativum	United Kingdom	7751309	7357	0.61	7276	7	Temperate broadleaf and mixed forests
2515154116	Rhizobium leguminosarum bv. viciae Ps8	this study	Alphaproteobacteria	Pisum sativum	United Kingdom	7552979	7359	0.61	7282	69	Temperate broadleaf and mixed forests
2516653047	Rhizobium leguminosarum bv. viciae TOM	this study	Alphaproteobacteria	Pisum sativum	Turkey	7357668	7255	0.61	7163	6	Temperate broadleaf and mixed forests
2515154113	Rhizobium leguminosarum bv. viciae Vc2	this study	Alphaproteobacteria	Vicia cracca	United Kingdom	7807172	7602	0.61	7478	142	Temperate broadleaf and mixed forests
2515154114	Rhizobium leguminosarum bv. viciae Vh3	this study	Alphaproteobacteria	Vicia hirsuta	United Kingdom	7848616	7647	0.61	7576	87	Temperate broadleaf and mixed forests
2529292951	Rhizobium leguminosarum CCGE 510	Servín-Garcidueñas LE, Rogel MA, Ormeño-Orrillo E, Delgado-Salinas A, Martínez-Romero J, Sánchez F, Martínez-Romero E. Genome sequence of Rhizobium sp. strain CCGE510, a symbiont isolated from nodules of the endangered wild bean Phaseolus albescens. (2012). J Bacteriol. 194(22):6310-1.	Alphaproteobacteria	Phaseolus albescens	Mexico	6916614	6642	0.61	6595	142	Tropical and subtropical coniferous forests
2524023209	Rhizobium leucaenae USDA 9039	this study	Alphaproteobacteria	Phaseolus vulgaris	Brazil	6679728	6564	0.59	6476	98	Tropical and subtropical grasslands, savannas and shrublands

2534682333	Rhizobium mesoamericanum STM 3625	Moulin, L., Mornico, D., Melkonian, R. & Klonowska, A. (2013). Draft genome sequence of Rhizobium mesoamericanum STM3625, a nitrogen-fixing symbiont of Mimosa pudica isolated in French Guiana (South America). Genome Announcements 1	Alphaproteobacteria	Mimosa pudica	French Guiana	6453427	6561	0.59	6494	92	Tropical and subtropical moist broadleaf forest
2513237088	Rhizobium mesoamericanum STM 6155	this study	Alphaproteobacteria	Mimosa pudica	New Caledonia	6927906	6926	0.59	6855	147	Tropical and subtropical dry broadleaf forest
2513237146	Rhizobium mongolense USDA 1844	this study	Alphaproteobacteria	Medicago ruthenica	China	7166346	7223	0.59	7144	96	Temperate grasslands, savannas and grasslands
2548876814	Rhizobium phaseoli Ch24-10	López-Guerrero, M. G., Ormeño-Orrillo, E., Velázquez, E., Rogel, M. A., Acosta, J. L., Gónzalez, V., Martínez, J. & Martínez-Romero, E. (2012). Rhizobium etli taxonomy revised with novel genomic data and analyses. Systematic and Applied Microbiology 35, 353-358	Alphaproteobacteria	Phaseolus vulgaris	Mexico	6622294	6593	0.61	6515	352	Tropical and subtropical grasslands, savannas and shrublands
2513237138	Rhizobium sp. OR 191	this study	Alphaproteobacteria	Medicago sativa	USA	7368160	7704	0.6	7617	240	Temperate coniferous forests
2519899620	Rhizobium sp. Pop5	unpublished	Alphaproteobacteria	Phaseolus vulgaris	Mexico	6499161	7071	0.61	7021	1413	
2513237144	Rhizobium sullae WSM1592	this study	Alphaproteobacteria	Hedysarum coronarium	Sardinia	7530820	7526	0.6	7453	118	Mediterranean forests, woodlands and scrub
2524023199	Rhizobium tropici CIAT 899	Ormeño-Orrillo et al. (2012) Genomic basis of broad host range and environmental adaptability of Rhizobium tropici CIAT 899 and Rhizobium sp. PRF 81 which are used in inoculants for common bean (Phaseolus vulgaris L.) BMC Genomics 13:735	Alphaproteobacteria	Phaseolus vulgaris	Colombia	6686334	6494	0.59	6432	4	Tropical and subtropical dry broadleaf forest
2545824643	Rhizobium tropici PRF 81	Ormeño-Orrillo et al. (2012) Genomic basis of broad host range and environmental adaptability of Rhizobium tropici CIAT 899 and Rhizobium sp. PRF 81 which are used in inoculants for common bean (Phaseolus vulgaris L.) BMC Genomics 13:735	Alphaproteobacteria	Phaseolus vulgaris	Brazil	7083871	6331	0.6	6271	96	Tropical and subtropical grasslands, savannas and shrublands

Supplementary Table 2. General genome metrics for 163 RNB strains analyzed in this study.

IMG taxon_oid	Status	Genome Name / Sample Name	Class	Order	Family	Genome Size	Gene Count	Scaffold Count	GC	CDS Count	RNA Count	rRNA Count	tRNA Count	w/ Func Pred Count	w/ Func Pred %	w/o function prediction	w/o function prediction %	COG Count	COG %	Pfam Count	Pfam %	TIGRfam Count	TIGRfam %	KO Count	KO %	Signal Peptide Count	Signal Peptide %	Biosynthetic Cluster Gene Count	Biosynth Cluster Gene %	Biosynth Cluster Count
2534682333	Permanent Draft	Rhizobium mesoamericanum STM3625	Alphaproteobacteria	Rhizobiales	Rhizobiaceae	6,453,427	6,561	92	0.59	6494	67	3	45	5191	79.12	1303	19.86	4307	65.65	5085	77.5	1526	23.26	2908	44.32	494	7.53	385	5.87	43
2513237164	Permanent Draft	Mesorhizobium loti CJ3sym	Alphaproteobacteria	Rhizobiales	Phyllobacteriaceae	7,563,725	7,401	70	0.62	7331	70	4	55	5682	76.77	1649	22.28	5074	68.56	5960	80.53	1644	22.21	3262	44.08	649	8.77	478	6.46	50
637000038	Finished	Bradyrhizobium japonicum USDA 110	Alphaproteobacteria	Rhizobiales	Bradyrhizobiaceae	9,105,828	8,402	1	0.64	8317	85	3	51	4530	53.92	3787	45.07	5329	63.43	6727	80.06	1856	22.09	3727	44.36	695	8.27	676	8.05	67
2515154189	Permanent Draft	Burkholderia nodosa DSM 21604	Betaproteobacteria	Burkholderiales	Burkholderiaceae	9,629,924	8,863	114	0.64	8777	86	8	52	7227	81.54	1550	17.49	6171	69.63	7400	83.49	2191	24.72	4044	45.63	861	9.71	712	8.03	66
2513237098	Permanent Draft	Bradyrhizobium elkanii WSM2783	Alphaproteobacteria	Rhizobiales	Bradyrhizobiaceae	9,902,361	9,734	217	0.62	9641	93	12	57	6876	70.64	2765	28.41	5739	58.96	7226	74.23	1931	19.84	3776	38.79	976	10.03	766	7.87	82
644736401	Finished	Rhizobium leguminosarum bv. trifolii WSM1325	Alphaproteobacteria	Rhizobiales	Rhizobiaceae	7,418,122	7,292	6	0.61	7232	60	9	51	5184	71.09	2048	28.09	5011	68.72	5984	82.06	1627	22.31	3377	46.31	652	8.94	397	5.44	36
2517572023	Finished	Ensifer fredii HH103	Alphaproteobacteria	Rhizobiales	Rhizobiaceae	7,221,188	6,851	10	0.62	6789	62	9	53	5627	82.13	1162	16.96	4704	68.66	5566	81.24	1585	23.14	3053	44.56	524	7.65	358	5.23	38
2516653047	Permanent Draft	Rhizobium leguminosarum bv. viciae TOM	Alphaproteobacteria	Rhizobiales	Rhizobiaceae	7,357,668	7,255	6	0.61	7163	92	9	57	5757	79.35	1406	19.38	5178	71.37	5985	82.49	1619	22.32	3410	47	631	8.7	417	5.75	39
2513237162	Permanent Draft	Rhizobium leguminosarum bv. viciae GB30	Alphaproteobacteria	Rhizobiales	Rhizobiaceae	7,468,464	7,302	78	0.61	7227	75	6	49	5816	79.65	1411	19.32	5182	70.97	6025	82.51	1616	22.13	3443	47.15	634	8.68	470	6.44	44
2529292711	Permanent Draft	Rhizobium etli CNPAF512	Alphaproteobacteria	Rhizobiales	Rhizobiaceae	6,726,123	7,586	402	0.61	7586	0	0	0	4438	58.5	3148	41.5	3809	50.21	4415	58.2	1300	17.14	2556	33.69	406	5.35	236	3.11	29
2524023228	Permanent Draft	Bradyrhizobium sp. Th.b2	Alphaproteobacteria	Rhizobiales	Bradyrhizobiaceae	10,118,060	9,917	266	0.63	9809	108	3	64	7023	70.82	2786	28.09	5576	56.23	7185	72.45	1875	18.91	3649	36.8	978	9.86	713	7.19	71
2517093000	Permanent Draft	Rhizobium leguminosarum bv. trifolii SRDI943	Alphaproteobacteria	Rhizobiales	Rhizobiaceae	7,412,387	7,406	5	0.61	7317	89	9	58	5823	78.63	1494	20.17	5171	69.82	6032	81.45	1651	22.29	3391	45.79	631	8.52	398	5.37	37
2512875026	Permanent Draft	Ensifer medicae WSM1115	Alphaproteobacteria	Rhizobiales	Rhizobiaceae	6,861,065	6,872	7	0.61	6789	83	9	56	5241	76.27	1548	22.53	4639	67.51	5422	78.9	1567	22.8	3092	44.99	571	8.31	318	4.63	32
2511231207	Finished	Bradyrhizobium japonicum USDA 6	Alphaproteobacteria	Rhizobiales	Bradyrhizobiaceae	9,207,384	8,886	1	0.64	8829	57	6	51	6692	75.31	2137	24.05	5509	62	6819	76.74	1816	20.44	3715	41.81	901	10.14	634	7.13	61
2513237138	Permanent Draft	Rhizobium sp. OR 191	Alphaproteobacteria	Rhizobiales	Rhizobiaceae	7,368,160	7,704	240	0.6	7617	87	6	49	5558	72.14	2059	26.73	4702	61.03	5866	76.14	1621	21.04	3169	41.13	576	7.48	387	5.02	39
642555152	Finished	Rhizobium etli CIAT 652	Alphaproteobacteria	Rhizobiales	Rhizobiaceae	6,448,048	6,132	4	0.61	6072	60	9	51	4091	66.72	1981	32.31	4337	70.73	4994	81.44	1532	24.98	2959	48.26	487	7.94	350	5.71	31
2545555834	Permanent Draft	Methylobacterium sp. WSM2598	Alphaproteobacteria	Rhizobiales	Methylobacteriaceae	8,130,841	7,667	85	0.71	7551	116	18	67	5559	72.51	1992	25.98	4463	58.21	5638	73.54	1717	22.39	2957	38.57	753	9.82	339	4.42	43
2551306089	Permanent Draft	Ensifer meliloti 1A42	Alphaproteobacteria	Rhizobiales	Rhizobiaceae	7,162,724	7,454	315	0.62	7360	94	6	60	5837	78.31	1523	20.43	4672	62.68	5920	79.42	1633	21.91	3012	40.41	567	7.61	405	5.43	43
2513237268	Permanent Draft	Bradyrhizobium sp. ORS285	Alphaproteobacteria	Rhizobiales	Bradyrhizobiaceae	7,602,254	6,842	301	0.65	6778	64	4	49	5417	79.17	1361	19.89	4563	66.69	5476	80.04	1681	24.57	3257	47.6	715	10.45	489	7.15	49
2512875016	Permanent Draft	Mesorhizobium loti R7A	Alphaproteobacteria	Rhizobiales	Phyllobacteriaceae	6,529,530	6,398	1	0.63	6323	75	8	58	5127	80.13	1196	18.69	4695	73.38	5332	83.34	1504	23.51	2978	46.55	565	8.83	510	7.97	44
2513237145	Permanent Draft	Bradyrhizobium elkanii USDA 3254	Alphaproteobacteria	Rhizobiales	Bradyrhizobiaceae	8,979,722	8,564	85	0.64	8487	77	3	53	6286	73.4	2201	25.7	5374	62.75	6635	77.48	1788	20.88	3529	41.21	900	10.51	702	8.2	65
643692032	Finished	Ensifer fredii NGR234	Alphaproteobacteria	Rhizobiales	Rhizobiaceae	6,891,900	6,437	3	0.62	6376	61	9	52	4788	74.38	1588	24.67	4578	71.12	5424	84.26	1580	24.55	3148	48.9	497	7.72	377	5.86	36
2528768022	Permanent Draft	Bradyrhizobium japonicum USDA 123	Alphaproteobacteria	Rhizobiales	Bradyrhizobiaceae	10,457,665	10,573	517	0.63	10476	97	3	57	7389	69.89	3087	29.2	5735	54.24	7580	71.69	1946	18.41	3749	35.46	1035	9.79	596	5.64	77
2515154122	Permanent Draft	Burkholderia sp. JPY251	Betaproteobacteria	Burkholderiales	Burkholderiaceae	8,609,520	7,983	122	0.63	7893	90	10	53	6330	79.29	1563	19.58	5352	67.04	6488	81.27	2032	25.45	3533	44.26	735	9.21	525	6.58	58
2513237087	Permanent Draft	Azorhizobium doebereinerae UFLA1-100	Alphaproteobacteria	Rhizobiales	Xanthobacteraceae	5,817,514	5,415	104	0.69	5347	68	8	49	4376	80.81	971	17.93	3820	70.54	4590	84.76	1521	28.09	2661	49.14	440	8.13	389	7.18	38
2513237096	Permanent Draft	Bradyrhizobium elkanii USDA 3259	Alphaproteobacteria	Rhizobiales	Bradyrhizobiaceae	8,722,461	8,327	101	0.64	8253	74	3	53	6153	73.89	2100	25.22	5259	63.16	6490	77.94	1764	21.18	3474	41.72	884	10.62	687	8.25	67
2509276022	Finished	Mesorhizobium australicum WSM2073	Alphaproteobacteria	Rhizobiales	Phyllobacteriaceae	6,200,534	6,080	1	0.63	6013	67	6	52	4876	80.2	1137	18.7	4451	73.21	5082	83.59	1510	24.84	2931	48.21	536	8.82	457	7.52	46
2508501009	Permanent Draft	Bradyrhizobium sp. WSM471	Alphaproteobacteria	Rhizobiales	Bradyrhizobiaceae	7,784,016	7,430	1	0.63	7372	58	3	47	5511	74.17	1861	25.05	4763	64.1	5762	77.55	1659	22.33	3221	43.35	834	11.22	509	6.85	51
649633066	Finished	Mesorhizobium ciceri bv. biserrulae WSM1271	Alphaproteobacteria	Rhizobiales	Phyllobacteriaceae	6,690,028	6,531	2	0.63	6470	61	6	52	4642	71.08	1828	27.99	4719	72.26	5398	82.65	1592	24.38	3132	47.96	597	9.14	428	6.55	44
2510065059	Permanent Draft	Mesorhizobium ciceri WSM4083	Alphaproteobacteria	Rhizobiales	Phyllobacteriaceae	6,847,884	6,662	42	0.63	6596	66	4	50	5262	78.99	1334	20.02	4734	71.06	5460	81.96	1610	24.17	3129	46.97	584	8.77	471	7.07	49
2524023207	Permanent Draft	Ensifer sp. USDA 6670, CC2017	Alphaproteobacteria	Rhizobiales	Rhizobiaceae	6,813,453	6,547	73	0.62	6465	82	4	57	5407	82.59	1058	16.16	4780	73.01	5485	83.78	1568	23.95	3198	48.85	589	9	394	6.02	38
2519899620	Permanent Draft	Rhizobium sp. Pop5	Alphaproteobacteria	Rhizobiales	Rhizobiaceae	6,499,161	7,071	1,413	0.61	7021	50	3	47	5945	84.08	1076	15.22	4068	57.53	5618	79.45	1484	20.99	2701	38.2	492	6.96	220	3.11	31
2517287029	Permanent Draft	Rhizobium leguminosarum bv. trifolii SRDI565	Alphaproteobacteria	Rhizobiales	Rhizobiaceae	6,905,599	6,836	7	0.61	6750	86	9	56	5338	78.09	1412	20.66	4736	69.28	5535	80.97	1555	22.75	3172	46.4	603	8.82	382	5.59	34
2513237083	Permanent Draft	Burkholderia mimosarum LMG 23256	Betaproteobacteria	Burkholderiales	Burkholderiaceae	8,410,967	7,886	268	0.64	7801	85	8	56	6112	77.5	1689	21.42	4974	63.07	6248	79.23	1957	24.82	3378	42.84	673	8.53	492	6.24	58
643348569	Finished	Rhizobium leguminosarum bv. trifolii WSM2304	Alphaproteobacteria	Rhizobiales	Rhizobiaceae	6,872,702	6,643	5	0.61	6581	62	9	53	4833	72.75	1748	26.31	4712	70.93	5544	83.46	1576	23.72	3231	48.64	599	9.02	405	6.1	38
2515075009	Permanent Draft	Rhizobium leguminosarum bv. viciae 248	Alphaproteobacteria	Rhizobiales	Rhizobiaceae	7,288,508	7,179	7	0.61	7090	89	9	51	5679	79.11	1411	19.65	5078	70.73	5880	81.91	1627	22.66	3363	46.84	644	8.97	425	5.92	42
2523533632	Finished	Ensifer meliloti GR4	Alphaproteobacteria	Rhizobiales	Rhizobiaceae	7,139,558	6,826	5	0.62	6707	119	9	55	5523	80.91	1184	17.35	4912	71.96	5592	81.92	1610	23.59	3204	46.94	590	8.64	367	5.38	40
2513237139	Permanent Draft	Bradyrhizobium japonicum USDA 4	Alphaproteobacteria	Rhizobiales	Bradyrhizobiaceae	8,737,671	8,243	97	0.64	8169	74	3	52	6058	73.49	2111	25.61	5152	62.5	6355	77.1	1773	21.51	3511	42.59	887	10.76	564	6.84	59
2515154107	Permanent Draft	Ensifer meliloti 4H41	Alphaproteobacteria	Rhizobiales	Rhizobiaceae	6,795,637	6,422	47	0.62	6350	72	4	50	5268	82.03	1082	16.85	4715	73.42	5435	84.63	1610	25.07	3150	49.05	553	8.61	399	6.21	42
2503198000	Finished	Mesorhizobium opportunistum WSM2075 (final version f	Alphaproteobacteria	Rhizobiales	Phyllobacteriaceae	6,884,444	6,747	1	0.63	6685	62	6	53	5380	79.74	1305	19.34	4874	72.24	5600	83	1580	23.42	3141	46.55	610	9.04	506	7.5	52
2547132261	Permanent Draft	Ensifer fredii GR64	Alphaproteobacteria	Rhizobiales	Rhizobiaceae	6,959,392	6,630	216	0.62	6551	79	3	49	5484	82.71	1067	16.09	4733	71.39	5569	84	1658	25.01	3177	47.92	537	8.1	367	5.54	40
2513237161	Permanent Draft	Bradyrhizobium sp. WSM2793	Alphaproteobacteria	Rhizobiales	Bradyrhizobiaceae	8,871,253	8,414	84	0.64	8349	65	3	50	6236	74.11	2113	25.11	5305	63.05	6534	77.66	1821	21.64	3554	42.24	914	10.86	659	7.83	63
2510065019	Finished	Rhizobium leguminosarum bv. trifolii WSM1689	Alphaproteobacteria	Rhizobiales	Rhizobiaceae	6,903,379	6,798	6	0.61	6709	89	9	51	5409	79.57	1300	19.12	4859	71.48	5618	82.64	1565	23.02	3236	47.6	591	8.69	382	5.62	35
2524023209	Permanent Draft	Rhizobium leucaenae USDA 9039	Alphaproteobacteria	Rhizobiales	Rhizobiaceae	6,679,728	6,564	98	0.59	6476	88	2	53	5343	81.4	1133	17.26	4581	69.79	5446	82.97	1604	24.44	3066	46.71	519	7.91	371	5.65	39
2513237102	Permanent Draft	Bradyrhizobium japonicum USDA 135	Alphaproteobacteria	Rhizobiales	Bradyrhizobiaceae	7,703,324	7,923	547	0.64	7858	65	3	46	5622	70.96	2236	28.22	4531	57.19	5995	75.67	1657	20.91	3134	39.56	773	9.76	504	6.36	65
2509276052	Permanent Draft	Rhizobium leguminosarum bv. trifolii CC278f	Alphaproteobacteria	Rhizobiales	Rhizobiaceae	8,729,608	8,665	7	0.61	8582	83	5	48	6849	79.04	1733	20	6007	69.32	7092	81.85	1819	20.99	3938	45.45	710	8.19	534	6.16	53
2513237092	Permanent Draft	Bradyrhizobium sp. WSM1743	Alphaproteobacteria	Rhizobiales	Bradyrhizobiaceae	8,341,956	7,983	163	0.63	7908	75	3	52	5720	71.65	2188	27.41	4824	60.43	6012	75.31	1710	21.42	3318	41.56	840	10.52	465	5.82	53
2510065056	Permanent Draft	Ensifer meliloti CIAM1775	Alphaproteobacteria	Rhizobiales	Rhizobiaceae	6,712,855	6,516	108	0.62	6440	76	3	53	5257	80.68	1183	18.16	4707	72.24	5442	83.52	1583	24.29	3132	48.07	559	8.58	394	6.05	40
2511231052	Permanent Draft	Ensifer meliloti AK58	Alphaproteobacteria	Rhizobiales	Rhizobiaceae	6,974,333	7,013	9	0.62	6934	79	3	49	5465	77.93	1469	20.95	4853	69.2	5649	80.55	1601	22.83	3155	44.99	580	8.27	398	5.68	42
2513237101	Permanent Draft	Bradyrhizobium elkanii WSM1741	Alphaproteobacteria	Rhizobiales	Bradyrhizobiaceae	7,952,346	7,649	69	0.62	7581	68	3	50	5747	75.13	1834	23.98	4863	63.58	6007	78.53	1681	21.98	3229	42.21	774	10.12	552	7.22	59
637000269	Finished	Ensifer meliloti 1021	Alphaproteobacteria	Rhizobiales	Rhizobiaceae	6,691,694	6,295	3	0.62	6212	83	9	55	3854	61.22	2358	37.46	4738	75.27	5378	85.43	1566	24.88	3211	51.01	503	7.99	355	5.64	39
2526164713	Permanent Draft	Burkholderia sp. JPY366	Betaproteobacteria	Burkholderiales	Burkholderiaceae	6,780,608	6,146	69	0.64	6070	76	6	47	5037	81.96	1033	16.81	4325	70.37	5168	84.09	1780	28.96	3035	49.38	633	10.3	341	5.55	33
2513237093	Permanent Draft	Rhizobium leguminosarum bv. phaseoli FA23	Alphaproteobacteria	Rhizobiales	Rhizobiaceae	7,545,552	7,285	66	0.61	7203	82	5	49	5836	80.11	1367	18.76	5204	71.43	6067	83.28	1664	22.84	3506	48.13	621	8.52	419	5.75	45
644736347	Finished	Cupriavidus taiwanensis LMG 19424	Betaproteobacteria	Burkholderiales	Burkholderiaceae	6,476,522	5,986	3	0.67	5897	89	15	63	4240	70.83	1657	27.68	4252	71.03	5022	83.9	1656	27.66	3037	50.74	625	10.44	431	7.2	42
2512875024	Permanent Draft	Mesorhizobium loti R88b	Alphaproteobacteria	Rhizobiales	Phyllobacteriaceae	7,195,110	7,016	1	0.62	6950	66	6	51	5556	79.19	1394	19.87	5015	71.48	5800	82.67	1639	23.36	3235	46.11	651	9.28	471	6.71	42
2513237137	Permanent Draft	Bradyrhizobium elkanii USDA 94	Alphaproteobacteria	Rhizobiales	Bradyrhizobiaceae	9,558,895	9,129	164	0.64	9057	72	3	51	6613	72.44	2444	26.77	5605	61.4	6976	76.42	1855	20.32	3683	40.34	913	10	809	8.86	77
2507262055	Permanent Draft	Bradyrhizobium sp. WSM1417	Alphaproteobacteria	Rhizobiales	Bradyrhizobiaceae	8,048,963	7,772	1	0.63	7695	77	3	52	5757	74.07	1938	24.94	4912	63.2	6011	77.34	1715	22.07	3331	42.86	872	11.22	596	7.67	54
2507525018	Permanent Draft	Rhizobium leguminosarum bv. trifolii CC283b	Alphaproteobacteria	Rhizobiales	Rhizobiaceae	7,923,462	7,731	11	0.61	7654	77	7	47	6085	78.71	1569	20.29	5326	68.89	6361	82.28	1680	21.73	3530	45.66	656	8.49	516	6.67	45
2519103084	Permanent Draft	Ensifer meliloti CCNWSX0020	Alphaproteobacteria	Rhizobiales	Rhizobiaceae	7,001,588	6,900	233	0.62	6847	53	5	48	5639	81.72	1208	17.51	4761	69	5597	81.12	1588	23.01	3159	45.78	523	7.58	341	4.94	36
2513237091	Permanent Draft	Ensifer meliloti RRI128	Alphaproteobacteria	Rhizobiales	Rhizobiaceae	6,900,273	6,770	156	0.62	6683	87	4	56	5336	78.82	1347	19.9	4717	69.68	5505	81.31	1605	23.71	3128	46.2	569	8.4	335	4.95	40
640427103	Finished	Bradyrhizobium sp. BTAi1	Alphaproteobacteria	Rhizobiales	Bradyrhizobiaceae	8,493,513	7,819	2	0.65	7741	78	7	52	4733	60.53	3008	38.47	5070	64.84	6289	80.43	1855	23.72	3715	47.51	723	9.25	536	6.86	52
2551306085	Permanent Draft	Ensifer meliloti AE608H	Alphaproteobacteria	Rhizobiales	Rhizobiaceae	7,347,181	7,285	223	0.62	7181	104	18	66	5733	78.7	1448	19.88	4990	68.5	5828	80	1674	22.98	3251	44.63	574	7.88	354	4.86	38
2548876814	Permanent Draft	Rhizobium phaseoli Ch24-10	Alphaproteobacteria	Rhizobiales	Rhizobiaceae	6,622,294	6,593	352	0.61	6515	78	4	49	5285	80.16	1230	18.66	4455	67.57	5369	81.43	1554	23.57	3005	45.58	550	8.34	341	5.17	31
2517487022	Permanent Draft	Ensifer medicae WSM4191	Alphaproteobacteria	Rhizobiales	Rhizobiaceae	7,227,575	7,099	6	0.61	7010	89	9	56	5650	79.59	1360	19.16	4998	70.4	5830	82.12	1635	23.03	3316	46.71	587	8.27	354	4.99	34
2513237351	Permanent Draft	Mesorhizobium alhagi CCNWXJ12-2	Alphaproteobacteria	Rhizobiales	Phyllobacteriaceae	6,968,952	7,244	375	0.63	7195	49	2	47	5525	76.27	1670	23.05	4407	60.84	5500	75.92	1583	21.85	2827	39.03	560	7.73	302	4.17	32
2518645580	Finished	Burkholderia phenoliruptrix BR3459	Betaproteobacteria	Burkholderiales	Burkholderiaceae	7,651,131	6,605	3	0.63	6525	80	18	62	5591	84.65	934	14.14	4753	71.96	5617	85.04	1886	28.55	3332	50.45	555	8.4	470	7.12	41
648276728	Finished	Ensifer meliloti BL225C	Alphaproteobacteria	Rhizobiales	Rhizobiaceae	6,968,865	6,769	158	0.62	6712	57	5	52	5094	75.25	1618	23.9	4823	71.25	5631	83.19	1607	23.74	3158	46.65	580	8.57	365	5.39	39
2509276018	Permanent Draft	Mesorhizobium ciceri CMG6	Alphaproteobacteria	Rhizobiales	Phyllobacteriaceae	6,910,194	6,718	90	0.62	6648	70	6	52	5380	80.08	1268	18.87	4830	71.9	5587	83.16	1610	23.97	3170	47.19	589	8.77	465	6.92	45
2513237090	Permanent Draft	Mesorhizobium sp. WSM3224	Alphaproteobacteria	Rhizobiales	Phyllobacteriaceae	7,096,802	6,951	72	0.63	6880	71	6	52	5505	79.2	1375	19.78	4901	70.51	5724	82.35	1612	23.19	3129	45.02	586	8.43	413	5.94	39
2513237153	Permanent Draft	Bradyrhizobium sp. WSM2254	Alphaproteobacteria	Rhizobiales	Bradyrhizobiaceae	9,086,516	8,597	100	0.64	8528	69	3	54	6666	77.54	1862	21.66	5600	65.14	6790	78.98	1845	21.46	3741	43.52	939	10.92	660	7.68	56
2508501123	Permanent Draft	Mesorhizobium sp. WSM3626	Alphaproteobacteria	Rhizobiales	Phyllobacteriaceae	6,283,661	6,243	106	0.63	6172	71	6	53	4881	78.18	1291	20.68	4367	69.95	5115	81.93	1477	23.66	2835	45.41	526	8.43	371	5.94	38
2524023205	Permanent Draft	Bradyrhizobium sp. Cp5.3	Alphaproteobacteria	Rhizobiales	Bradyrhizobiaceae	8,918,781	8,545	148	0.63	8465	80	5	50	6463	75.63	2002	23.43	5264	61.6	6595	77.18	1862	21.79	3569	41.77	971	11.36	694	8.12	67
646564515	Finished	Burkholderia sp. CCGE1002	Betaproteobacteria	Burkholderiales	Burkholderiaceae	7,884,858	7,358	4	0.63	7261	97	21	73	5200	70.67	2061	28.01	4808	65.34	5907	80.28	1941	26.38	3305	44.92	676	9.19	406	5.52	40
2508501100	Permanent Draft	Rhizobium leguminosarum bv. trifolii WSM2297	Alphaproteobacteria	Rhizobiales	Rhizobiaceae	7,999,455	7,827	4	0.61	7746	81	5	48	5768	73.69	1978	25.27	5589	71.41	6505	83.11	1788	22.84	3700	47.27	652	8.33	431	5.51	39
2551306091	Permanent Draft	Ensifer meliloti C0431A	Alphaproteobacteria	Rhizobiales	Rhizobiaceae	7,086,830	7,449	401	0.62	7373	76	4	50	5836	78.35	1537	20.63	4527	60.77	5936	79.69	1583	21.25	2890	38.8	547	7.34	318	4.27	35
2515154115	Permanent Draft	Rhizobium leguminosarum bv. viciae 128C53	Alphaproteobacteria	Rhizobiales	Rhizobiaceae	7,609,235	7,396	72	0.61	7322	74	0	48	5932	80.21	1390	18.79	5270	71.25	6162	83.32	1666	22.53	3489	47.17	638	8.63	450	6.08	46
2508501042	Permanent Draft	Bradyrhizobium sp. WSM1253	Alphaproteobacteria	Rhizobiales	Bradyrhizobiaceae	8,719,808	8,498	2	0.63	8432	66	3	49	5692	66.98	2740	32.24	5310	62.49	6484	76.3	1835	21.59	3536	41.61	948	11.16	639	7.52	53
2513237086	Permanent Draft	Ensifer meliloti MVII-I	Alphaproteobacteria	Rhizobiales	Rhizobiaceae	7,265,287	7,213	220	0.62	7133	80	3	52	5566	77.17	1567	21.72	4896	67.88	5799	80.4	1651	22.89	3217	44.6	590	8.18	407	5.64	46
2513237089	Permanent Draft	Ensifer medicae Di28	Alphaproteobacteria	Rhizobiales	Rhizobiaceae	6,553,624	6,469	104	0.61	6394	75	3	53	5089	78.67	1305	20.17	4493	69.45	5263	81.36	1535	23.73	3035	46.92	534	8.25	292	4.51	33
2513237082	Permanent Draft	Burkholderia mimosarum STM3621	Betaproteobacteria	Burkholderiales	Burkholderiaceae	8,640,282	8,145	268	0.64	8062	83	8	57	6277	77.07	1785	21.92	5104	62.66	6428	78.92	2011	24.69	3451	42.37	684	8.4	430	5.28	48
2508501050	Permanent Draft	Microvirga lupini Lut6	Alphaproteobacteria	Rhizobiales	Methylobacteriaceae	9,633,614	10,951	160	0.62	10864	87	3	59	6946	63.43	3918	35.78	5173	47.24	7343	67.05	1748	15.96	3297	30.11	768	7.01	638	5.83	60
2509276021	Permanent Draft	Rhizobium leguminosarum bv. trifolii WSM597	Alphaproteobacteria	Rhizobiales	Rhizobiaceae	7,634,384	7,481	2	0.61	7394	87	6	46	5932	79.29	1462	19.54	5280	70.58	6150	82.21	1708	22.83	3517	47.01	634	8.47	371	4.96	37
2513237097	Permanent Draft	Bradyrhizobium japonicum USDA 6	Alphaproteobacteria	Rhizobiales	Bradyrhizobiaceae	9,131,141	8,736	87	0.64	8655	81	6	57	6426	73.56	2229	25.52	5482	62.75	6758	77.36	1817	20.8	3666	41.96	959	10.98	634	7.26	66
641228476	Finished	Azorhizobium caulinodans ORS 571	Alphaproteobacteria	Rhizobiales	Xanthobacteraceae	5,369,772	4,781	1	0.67	4718	63	9	53	3611	75.53	1107	23.15	3629	75.9	4178	87.39	1489	31.14	2556	53.46	308	6.44	308	6.44	29
2516653046	Permanent Draft	Ensifer meliloti BO21CC	Alphaproteobacteria	Rhizobiales	Rhizobiaceae	6,989,037	6,907	3	0.62	6805	102	9	58	5451	78.92	1354	19.6	4892	70.83	5616	81.31	1612	23.34	3166	45.84	611	8.85	396	5.73	41
2551306148	Permanent Draft	Ensifer meliloti H1	Alphaproteobacteria	Rhizobiales	Rhizobiaceae	6,923,151	7,084	397	0.62	6990	94	9	56	5623	79.38	1367	19.3	4576	64.6	5717	80.7	1592	22.47	2966	41.87	566	7.99	334	4.71	39
2524023212	Permanent Draft	Cupriavidus sp. AMP6	Betaproteobacteria	Burkholderiales	Burkholderiaceae	7,579,563	7,130	260	0.65	7033	97	5	54	5722	80.25	1311	18.39	4791	67.19	5837	81.87	1806	25.33	3327	46.66	681	9.55	538	7.55	63
641522639	Finished	Methylobacterium sp. 4-46	Alphaproteobacteria	Rhizobiales	Methylobacteriaceae	7,737,025	7,125	3	0.72	7043	82	18	63	4559	63.99	2484	34.86	4260	59.79	5390	75.65	1677	23.54	2892	40.59	725	10.18	364	5.11	39
2516653048	Permanent Draft	Bradyrhizobium genosp. SA-4 CB756	Alphaproteobacteria	Rhizobiales	Bradyrhizobiaceae	9,826,880	9,456	2	0.64	9373	83	3	50	6951	73.51	2422	25.61	5957	63	7269	76.87	1931	20.42	3939	41.66	1121	11.85	730	7.72	67
2513237141	Permanent Draft	Bradyrhizobium sp. TV2a.2	Alphaproteobacteria	Rhizobiales	Bradyrhizobiaceae	8,496,279	8,181	87	0.62	8109	72	3	57	5970	72.97	2139	26.15	4871	59.54	6080	74.32	1718	21	3260	39.85	866	10.59	665	8.13	70
2513237159	Permanent Draft	Rhizobium giardinii bv. giardinii H152	Alphaproteobacteria	Rhizobiales	Rhizobiaceae	6,810,126	6,691	190	0.61	6618	73	2	48	5313	79.41	1305	19.5	4585	68.52	5518	82.47	1628	24.33	3071	45.9	556	8.31	392	5.86	42
2516653085	Permanent Draft	Rhizobium leguminosarum bv. phaseoli 4292	Alphaproteobacteria	Rhizobiales	Rhizobiaceae	7,346,596	7,193	5	0.61	7109	84	9	53	5786	80.44	1323	18.39	5163	71.78	6018	83.66	1653	22.98	3430	47.69	622	8.65	458	6.37	41
2513237104	Permanent Draft	Bradyrhizobium sp. EC3.3	Alphaproteobacteria	Rhizobiales	Bradyrhizobiaceae	10,034,502	9,729	154	0.63	9656	73	3	52	7056	72.53	2600	26.72	5672	58.3	7190	73.9	1903	19.56	3863	39.71	1034	10.63	618	6.35	62
2510065057	Permanent Draft	Ensifer meliloti WSM1022	Alphaproteobacteria	Rhizobiales	Rhizobiaceae	6,649,661	6,398	121	0.62	6323	75	5	51	5171	80.82	1152	18.01	4646	72.62	5340	83.46	1586	24.79	3093	48.34	563	8.8	336	5.25	39
2513237136	Permanent Draft	Bradyrhizobium sp. USDA 3384	Alphaproteobacteria	Rhizobiales	Bradyrhizobiaceae	9,800,269	9,313	51	0.64	9240	73	3	50	7018	75.36	2222	23.86	5835	62.65	7158	76.86	1913	20.54	3865	41.5	1106	11.88	674	7.24	68
2513237160	Permanent Draft	Ensifer medicae WSM244	Alphaproteobacteria	Rhizobiales	Rhizobiaceae	6,650,282	6,495	91	0.61	6427	68	4	48	5153	79.34	1274	19.62	4567	70.32	5311	81.77	1548	23.83	3060	47.11	536	8.25	311	4.79	33
643348564	Finished	Methylobacterium nodulans ORS 2060	Alphaproteobacteria	Rhizobiales	Methylobacteriaceae	8,839,022	8,885	8	0.68	8791	94	21	73	5169	58.18	3622	40.77	4755	53.52	6138	69.08	1781	20.05	3070	34.55	729	8.2	619	6.97	57
642555112	Finished	Burkholderia phymatum STM815	Betaproteobacteria	Burkholderiales	Burkholderiaceae	8,676,562	7,574	4	0.62	7496	78	16	62	5615	74.14	1881	24.83	5165	68.19	6296	83.13	2063	27.24	3557	46.96	701	9.26	499	6.59	49
2513237150	Permanent Draft	Cupriavidus taiwanensis STM6018	Betaproteobacteria	Burkholderiales	Burkholderiaceae	6,553,639	5,925	80	0.67	5864	61	7	48	4891	82.55	973	16.42	4324	72.98	5055	85.32	1702	28.73	3056	51.58	663	11.19	382	6.45	48
2517572143	Permanent Draft	Bradyrhizobium elkanii USDA 76	Alphaproteobacteria	Rhizobiales	Bradyrhizobiaceae	9,484,767	9,151	2	0.64	9060	91	3	51	6881	75.19	2179	23.81	5665	61.91	7034	76.87	1862	20.35	3707	40.51	864	9.44	789	8.62	72
2562617130	Finished	Ensifer meliloti 2011	Alphaproteobacteria	Rhizobiales	Rhizobiaceae	6,693,185	8,251	3	0.62	6312	1939	9	55	5432	65.83	880	10.67	4799	58.16	5392	65.35	1579	19.14	3195	38.72	527	6.39	349	4.23	38
2551306086	Permanent Draft	Ensifer meliloti AK11	Alphaproteobacteria	Rhizobiales	Rhizobiaceae	6,843,938	6,988	424	0.62	6902	86	7	51	5618	80.39	1284	18.37	4583	65.58	5696	81.51	1604	22.95	3001	42.95	535	7.66	350	5.01	41

2513237088	Permanent Draft	Rhizobium mesoamericanum STM6155	Alphaproteobacteria	Rhizobiales	Rhizobiaceae	6,927,906	6,926	147	0.59	6855	71	3	47	5281	76.25	1574	22.73	4585	66.2	5526	79.79	1606	23.19	3081	44.48	538	7.77	433	6.25	49
640753051	Finished	Ensifer medicae WSM419	Alphaproteobacteria	Rhizobiales	Rhizobiaceae	6,817,576	6,599	4	0.61	6518	81	9	53	4671	70.78	1847	27.99	4647	70.42	5454	82.65	1577	23.9	3127	47.39	541	8.2	339	5.14	34
2551306087	Permanent Draft	Ensifer meliloti A0643DD	Alphaproteobacteria	Rhizobiales	Rhizobiaceae	7,351,905	7,849	678	0.62	7755	94	6	56	5991	76.33	1764	22.47	4609	58.72	6084	77.51	1648	21	2964	37.76	580	7.39	369	4.7	46
2545824643	Permanent Draft	Rhizobium tropici PRF 81	Alphaproteobacteria	Rhizobiales	Rhizobiaceae	7,083,871	6,331	96	0.6	6271	60	9	51	5199	82.12	1072	16.93	4466	70.54	5183	81.87	1492	23.57	2988	47.2	435	6.87	342	5.4	34
2513237163	Permanent Draft	Cupriavidus sp. gt. E UYPR2.512	Betaproteobacteria	Burkholderiales	Burkholderiaceae	7,858,949	7,487	365	0.65	7411	76	6	56	5663	75.64	1748	23.35	4807	64.2	5959	79.59	1804	24.1	3323	44.38	696	9.3	419	5.6	45
2513237312	Permanent Draft	Bradyrhizobium sp. STM 3809	Alphaproteobacteria	Rhizobiales	Bradyrhizobiaceae	7,311,748	6,699	803	0.66	6637	62	3	46	5192	77.5	1445	21.57	4237	63.25	5217	77.88	1558	23.26	3060	45.68	693	10.34	285	4.25	41
2516653074	Permanent Draft	Burkholderia sp. WSM4176	Betaproteobacteria	Burkholderiales	Burkholderiaceae	9,065,247	8,497	13	0.63	8369	128	21	66	6416	75.51	1953	22.98	5491	64.62	6766	79.63	2034	23.94	3649	42.94	738	8.69	648	7.63	56
2513237103	Permanent Draft	Rhizobium leguminosarum bv. viciae VF39	Alphaproteobacteria	Rhizobiales	Rhizobiaceae	7,647,401	7,469	89	0.61	7392	77	2	48	5948	79.64	1444	19.33	5255	70.36	6157	82.43	1664	22.28	3492	46.75	643	8.61	490	6.56	59
2508501124	Permanent Draft	Burkholderia dilworthii WSM3556	Betaproteobacteria	Burkholderiales	Burkholderiaceae	7,679,067	7,123	140	0.62	7059	64	4	47	5433	76.27	1626	22.83	4704	66.04	5730	80.44	1831	25.71	3186	44.73	642	9.01	426	5.98	39
2519103088	Permanent Draft	Bradyrhizobium sp. ORS 375	Alphaproteobacteria	Rhizobiales	Bradyrhizobiaceae	7,859,468	7,143	497	0.65	7078	65	3	51	5546	77.64	1532	21.45	4565	63.91	5586	78.2	1687	23.62	3253	45.54	732	10.25	469	6.57	53
2513237143	Permanent Draft	Ensifer meliloti Mlalz-1	Alphaproteobacteria	Rhizobiales	Rhizobiaceae	6,664,116	6,388	99	0.62	6314	74	2	52	5192	81.28	1122	17.56	4659	72.93	5357	83.86	1579	24.72	3080	48.22	555	8.69	355	5.56	37
2515154113	Permanent Draft	Rhizobium leguminosarum bv. viciae Vc2	Alphaproteobacteria	Rhizobiales	Rhizobiaceae	7,807,172	7,602	142	0.61	7478	124	3	53	6052	79.61	1426	18.76	5348	70.35	6279	82.6	1674	22.02	3556	46.78	654	8.6	471	6.2	47
2510065045	Permanent Draft	Burkholderia sprentiae WSM5005	Betaproteobacteria	Burkholderiales	Burkholderiaceae	7,761,063	7,223	8	0.63	7147	76	7	58	5506	76.23	1641	22.72	4762	65.93	5810	80.44	1890	26.17	3253	45.04	687	9.51	491	6.8	46
2516653077	Permanent Draft	Rhizobium leguminosarum bv. viciae WSM1481	Alphaproteobacteria	Rhizobiales	Rhizobiaceae	7,555,578	7,464	6	0.61	7374	90	9	55	5973	80.02	1401	18.77	5355	71.74	6183	82.84	1674	22.43	3513	47.07	647	8.67	478	6.4	43
2513237084	Permanent Draft	Rhizobium leguminosarum bv. viciae UPM1131	Alphaproteobacteria	Rhizobiales	Rhizobiaceae	7,231,967	6,951	41	0.61	6870	81	5	46	5635	81.07	1235	17.77	5064	72.85	5846	84.1	1637	23.55	3376	48.57	633	9.11	461	6.63	45
2513237146	Permanent Draft	Rhizobium mongolense USDA 1844	Alphaproteobacteria	Rhizobiales	Rhizobiaceae	7,166,346	7,223	96	0.59	7144	79	5	48	5433	75.22	1711	23.69	4686	64.88	5643	78.13	1571	21.75	3165	43.82	556	7.7	409	5.66	36
2517572076	Permanent Draft	Mesorhizobium loti NZP2037	Alphaproteobacteria	Rhizobiales	Phyllobacteriaceae	7,462,792	7,388	1	0.63	7318	70	4	52	5985	81.01	1333	18.04	5271	71.35	6121	82.85	1662	22.5	3367	45.57	654	8.85	572	7.74	51
2534681796	Permanent Draft	Rhizobium sp. CCGE 502	Alphaproteobacteria	Rhizobiales	Rhizobiaceae	7,146,037	6,898	80	0.59	6844	54	4	50	5657	82.01	1187	17.21	4685	67.92	5586	80.98	1617	23.44	3097	44.9	533	7.73	298	4.32	36
2513237144	Permanent Draft	Rhizobium sullae WSM1592	Alphaproteobacteria	Rhizobiales	Rhizobiaceae	7,530,820	7,526	118	0.6	7453	73	4	47	5907	78.49	1546	20.54	5148	68.4	6174	82.04	1676	22.27	3417	45.4	659	8.76	538	7.15	47
2517093001	Permanent Draft	Bradyrhizobium japonicum USDA 124	Alphaproteobacteria	Rhizobiales	Bradyrhizobiaceae	9,002,274	8,621	123	0.64	8509	112	3	52	6253	72.53	2256	26.17	5338	61.92	6595	76.5	1802	20.9	3562	41.32	966	11.21	540	6.26	60
2524023210	Permanent Draft	Bradyrhizobium sp. Ai1a-2	Alphaproteobacteria	Rhizobiales	Bradyrhizobiaceae	9,029,266	8,584	246	0.63	8482	102	3	80	6449	75.13	2033	23.68	5111	59.54	6590	76.77	1827	21.28	3445	40.13	837	9.75	837	9.75	90
2551306092	Permanent Draft	Ensifer meliloti AK75	Alphaproteobacteria	Rhizobiales	Rhizobiaceae	6,992,595	7,636	700	0.62	7550	86	6	59	5962	78.08	1588	20.8	4499	58.92	6046	79.18	1587	20.78	2880	37.72	544	7.12	311	4.07	35
650716086	Finished	Ensifer meliloti AK83, DSM 23913	Alphaproteobacteria	Rhizobiales	Rhizobiaceae	7,140,471	7,022	5	0.62	6954	68	9	56	4897	69.74	2057	29.29	4825	68.71	5633	80.22	1602	22.81	3139	44.7	563	8.02	381	5.43	39
2512047086	Permanent Draft	Ensifer arboris LMG 14919	Alphaproteobacteria	Rhizobiales	Rhizobiaceae	6,850,303	6,545	7	0.62	6461	84	9	54	5291	80.84	1170	17.88	4743	72.47	5447	83.22	1638	25.03	3190	48.74	588	8.98	364	5.56	42
2513237166	Permanent Draft	Burkholderia sp. UYPR1.413	Betaproteobacteria	Burkholderiales	Burkholderiaceae	10,373,764	9,836	336	0.62	9759	77	8	50	7469	75.94	2290	23.28	6103	62.05	7650	77.78	2306	23.44	4048	41.15	934	9.5	471	4.79	52
2508501128	Permanent Draft	Bradyrhizobium sp. ARR65	Alphaproteobacteria	Rhizobiales	Bradyrhizobiaceae	8,613,869	8,310	139	0.62	8246	64	3	49	5921	71.25	2325	27.98	4962	59.71	6239	75.08	1744	20.99	3386	40.75	798	9.6	606	7.29	63
2551306090	Permanent Draft	Ensifer meliloti A0641M	Alphaproteobacteria	Rhizobiales	Rhizobiaceae	7,953,713	8,520	451	0.62	8412	108	9	66	6415	75.29	1997	23.44	5038	59.13	6514	76.46	1798	21.1	3219	37.78	596	7	409	4.8	42
2513237156	Permanent Draft	Ensifer medicae WSM1369	Alphaproteobacteria	Rhizobiales	Rhizobiaceae	6,402,557	6,735	307	0.61	6656	79	3	48	4994	74.15	1662	24.68	4179	62.05	5188	77.03	1542	22.9	2842	42.2	508	7.54	289	4.29	33
2509276044	Permanent Draft	Rhizobium leguminosarum bv. viciae WSM1455	Alphaproteobacteria	Rhizobiales	Rhizobiaceae	7,557,514	7,494	3	0.61	7418	76	6	48	5464	72.91	1954	26.07	5359	71.51	6176	82.41	1674	22.34	3505	46.77	649	8.66	461	6.15	42
2513237147	Permanent Draft	Mesorhizobium loti USDA 3471	Alphaproteobacteria	Rhizobiales	Phyllobacteriaceae	7,023,073	6,785	50	0.63	6714	71	6	52	5443	80.22	1271	18.73	4913	72.41	5669	83.55	1609	23.71	3207	47.27	607	8.95	412	6.07	44
2516143018	Permanent Draft	Ensifer sp. BR816	Alphaproteobacteria	Rhizobiales	Rhizobiaceae	6,951,533	6,643	3	0.62	6554	89	9	56	5419	81.57	1135	17.09	4812	72.44	5586	84.09	1699	25.58	3232	48.65	561	8.44	303	4.56	31
2524023199	Finished	Rhizobium tropici CIAT899	Alphaproteobacteria	Rhizobiales	Rhizobiaceae	6,686,334	6,494	4	0.59	6432	62	9	53	5390	83	1042	16.05	4735	72.91	5364	82.6	1574	24.24	3146	48.44	499	7.68	340	5.24	36
637000159	Finished	Mesorhizobium loti MAFF303099	Alphaproteobacteria	Rhizobiales	Phyllobacteriaceae	7,596,297	7,356	3	0.63	7272	84	6	53	4100	55.74	3172	43.12	5144	69.93	5942	80.78	1614	21.94	3267	44.41	494	6.72	531	7.22	49
2510461076	Permanent Draft	Rhizobium leguminosarum bv. trifolii TA1	Alphaproteobacteria	Rhizobiales	Rhizobiaceae	8,618,824	8,576	6	0.61	8493	83	5	57	6685	77.95	1808	21.08	5897	68.76	6944	80.97	1838	21.43	3886	45.31	727	8.48	517	6.03	48
2509276019	Permanent Draft	Ensifer sp. TW10	Alphaproteobacteria	Rhizobiales	Rhizobiaceae	6,802,256	6,546	57	0.62	6473	73	3	50	5080	77.6	1393	21.28	4481	68.45	5284	80.72	1572	24.01	2992	45.71	539	8.23	399	6.1	42
2513237305	Permanent Draft	Mesorhizobium amorphae CCNWGS0123	Alphaproteobacteria	Rhizobiales	Phyllobacteriaceae	7,293,571	7,136	274	0.63	7084	52	2	50	5800	81.28	1284	17.99	4836	67.77	5768	80.83	1589	22.27	3175	44.49	573	8.03	440	6.17	45
2508501114	Permanent Draft	Microvirga lotononidis WSM3557	Alphaproteobacteria	Rhizobiales	Methylobacteriaceae	7,082,538	7,040	18	0.63	6956	84	6	58	4774	67.81	2182	30.99	4382	62.24	5358	76.11	1559	22.14	2930	41.62	656	9.32	420	5.97	44
2510065076	Finished	Rhizobium leguminosarum bv. trifolii CB782	Alphaproteobacteria	Rhizobiales	Rhizobiaceae	6,703,653	6,559	4	0.61	6472	87	9	50	5288	80.62	1184	18.05	4756	72.51	5493	83.75	1594	24.3	3180	48.48	586	8.93	358	5.46	33
2515154134	Permanent Draft	Rhizobium gallicum bv. gallicum R602sp	Alphaproteobacteria	Rhizobiales	Rhizobiaceae	7,220,242	6,977	38	0.61	6900	77	2	53	5632	80.72	1268	18.17	5033	72.14	5828	83.53	1615	23.15	3356	48.1	618	8.86	405	5.8	40
2515154116	Permanent Draft	Rhizobium leguminosarum bv. viciae Ps8	Alphaproteobacteria	Rhizobiales	Rhizobiaceae	7,552,979	7,359	69	0.61	7282	77	2	51	5825	79.15	1457	19.8	5182	70.42	6056	82.29	1642	22.31	3423	46.51	642	8.72	476	6.47	43
639633055	Finished	Rhizobium leguminosarum bv. viciae 3841	Alphaproteobacteria	Rhizobiales	Rhizobiaceae	7,751,309	7,357	7	0.61	7276	81	9	52	5262	71.52	2014	27.38	5191	70.56	6163	83.77	1653	22.47	3550	48.25	574	7.8	540	7.34	52
2551306093	Permanent Draft	Ensifer meliloti C0438LL	Alphaproteobacteria	Rhizobiales	Rhizobiaceae	7,064,773	7,332	492	0.62	7237	95	6	55	5722	78.04	1515	20.66	4596	62.68	5812	79.27	1626	22.18	2978	40.62	566	7.72	368	5.02	42
2513237148	Permanent Draft	Bradyrhizobium japonicum USDA 38	Alphaproteobacteria	Rhizobiales	Bradyrhizobiaceae	9,608,975	9,258	107	0.64	9173	85	6	55	6641	71.73	2532	27.35	5626	60.77	6994	75.55	1859	20.08	3762	40.64	1012	10.93	728	7.86	80
2551306084	Permanent Draft	Ensifer meliloti 5A14	Alphaproteobacteria	Rhizobiales	Rhizobiaceae	8,942,552	8,870	217	0.62	8728	142	24	83	7029	79.24	1699	19.15	6098	68.75	7143	80.53	1996	22.5	3960	44.64	738	8.32	489	5.51	54
2508501127	Permanent Draft	Mesorhizobium sp. WSM2561	Alphaproteobacteria	Rhizobiales	Phyllobacteriaceae	7,037,543	6,910	111	0.62	6834	76	6	52	5245	75.9	1589	23	4523	65.46	5464	79.07	1546	22.37	3007	43.52	539	7.8	491	7.11	45
2515154112	Permanent Draft	Bradyrhizobium sp. WSM4349	Alphaproteobacteria	Rhizobiales	Bradyrhizobiaceae	8,294,334	8,114	104	0.63	8033	81	3	47	5857	72.18	2176	26.82	4919	60.62	6145	75.73	1719	21.19	3353	41.32	832	10.25	583	7.19	55
2515154114	Permanent Draft	Rhizobium leguminosarum bv. viciae Vh3	Alphaproteobacteria	Rhizobiales	Rhizobiaceae	7,848,616	7,647	87	0.61	7576	71	0	48	6089	79.63	1487	19.45	5419	70.86	6315	82.58	1643	21.49	3582	46.84	657	8.59	496	6.49	49
2513237085	Permanent Draft	Rhizobium leguminosarum bv. viciae UPM1137	Alphaproteobacteria	Rhizobiales	Rhizobiaceae	7,695,351	7,462	49	0.61	7390	72	3	49	5919	79.32	1471	19.71	5295	70.96	6146	82.36	1660	22.25	3520	47.17	675	9.05	501	6.71	47
640427104	Finished	Bradyrhizobium sp. ORS278	Alphaproteobacteria	Rhizobiales	Bradyrhizobiaceae	7,456,587	6,825	1	0.66	6752	73	6	50	4069	59.62	2683	39.31	4421	64.78	5452	79.88	1647	24.13	3269	47.9	636	9.32	516	7.56	47
2514885035	Finished	Ensifer fredii USDA 257	Alphaproteobacteria	Rhizobiales	Rhizobiaceae	7,032,323	6,855	20	0.62	6793	62	9	53	5903	86.11	890	12.98	4587	66.91	5392	78.66	1581	23.06	3137	45.76	478	6.97	326	4.76	35
2529292951	Permanent Draft	Rhizobium sp. CCGE 510	Alphaproteobacteria	Rhizobiales	Rhizobiaceae	6,916,614	6,642	142	0.61	6595	47	2	45	5583	84.06	1012	15.24	4747	71.47	5509	82.94	1605	24.16	3233	48.68	534	8.04	368	5.54	33
2513237140	Permanent Draft	Ensifer meliloti GVPV12	Alphaproteobacteria	Rhizobiales	Rhizobiaceae	7,076,289	6,806	106	0.62	6727	79	6	51	5461	80.24	1266	18.6	4811	70.69	5640	82.87	1655	24.32	3159	46.41	560	8.23	399	5.86	39
2513237165	Permanent Draft	Cupriavidus taiwanensis STM6070	Betaproteobacteria	Burkholderiales	Burkholderiaceae	6,771,773	6,182	107	0.67	6118	64	5	51	5140	83.14	978	15.82	4500	72.79	5324	86.12	1740	28.15	3153	51	677	10.95	377	6.1	45
2508501122	Permanent Draft	Ensifer sp. WSM1721	Alphaproteobacteria	Rhizobiales	Rhizobiaceae	6,292,184	6,011	68	0.62	5935	76	5	50	4927	81.97	1008	16.77	4339	72.18	4988	82.98	1535	25.54	2899	48.23	516	8.58	316	5.26	37
2513237094	Permanent Draft	Bradyrhizobium sp. WSM3983	Alphaproteobacteria	Rhizobiales	Bradyrhizobiaceae	8,789,602	8,469	70	0.63	8402	67	3	50	6481	76.53	1921	22.68	5368	63.38	6594	77.86	1863	22	3623	42.78	876	10.34	633	7.47	61
640427137	Finished	Rhizobium etli CFN 42, DSM 11541	Alphaproteobacteria	Rhizobiales	Rhizobiaceae	6,530,228	6,117	7	0.61	6038	79	9	50	4260	69.64	1778	29.07	4422	72.29	5177	84.63	1512	24.72	3101	50.69	469	7.67	382	6.24	32
651053067	Finished	Ensifer meliloti SM11	Alphaproteobacteria	Rhizobiales	Rhizobiaceae	7,173,736	7,156	3	0.62	7093	63	9	54	4598	64.25	2495	34.87	4928	68.87	5688	79.49	1635	22.85	3205	44.79	531	7.42	397	5.55	38
2513237095	Permanent Draft	Bradyrhizobium japonicum USDA 122	Alphaproteobacteria	Rhizobiales	Bradyrhizobiaceae	8,976,980	8,530	101	0.64	8451	79	3	52	6242	73.18	2209	25.9	5287	61.98	6542	76.69	1812	21.24	3591	42.1	968	11.35	640	7.5	66
2509276033	Permanent Draft	Rhizobium leguminosarum bv. trifolii WSM2012	Alphaproteobacteria	Rhizobiales	Rhizobiaceae	7,180,565	7,166	6	0.61	7080	86	10	52	5232	73.01	1848	25.79	5007	69.87	5892	82.22	1669	23.29	3353	46.79	615	8.58	368	5.14	36
2508501126	Permanent Draft	Mesorhizobium sp. WSM1293	Alphaproteobacteria	Rhizobiales	Phyllobacteriaceae	6,938,611	6,711	51	0.62	6640	71	6	53	5337	79.53	1303	19.42	4785	71.3	5540	82.55	1591	23.71	3142	46.82	599	8.93	465	6.93	44
2512047030	Draft	Burkholderia tuberum STM678	Betaproteobacteria	Burkholderiales	Burkholderiaceae	9,031,815	8,658	284	0.63	8534	124	38	67	6387	73.77	2147	24.8	5262	60.78	6768	78.17	1989	22.97	3520	40.66	659	7.61	622	7.18	62

Supplementary Table 3. List of NC genomes used for comparative analysis in this study.

IMG taxon_oid	Genome Name			
2515154138	Aurantimonas ureilytica DSM 18598			
651053055	Oligotropha carboxidovorans OM4			
2510065041	Oligotropha carboxidovorans OM5			
2516653076	Hyphomicrobium sp. 802			
2545824543	Hyphomicrobium sp. 99			
2516653063	Methyloferula stellata AR4			
638341134	Nitrobacter sp. Nb-311A			
2502376840	Prosthecomicrobium hirschii ATCC 27832			
639279312	Rhodopseudomonas palustris BisA53			
637000237	Rhodopseudomonas palustris BisB18			
637000238	Rhodopseudomonas palustris BisB5			
637000240	Rhodopseudomonas palustris HaA2			
2551306146	Afipia birgiae 34632			
2517572147	Amorphus coralli DSM 19760			
2523533557	Aurantimonas coralicida DSM 14790			
639857001	Fulvimarina pelagi HTCC2506			
2514752000	Nitratireductor aquibiodomus RA22 (Draft1)			
2519899619	Nitratireductor indicus C115			
648028050	Parvularcula bermudensis HTCC2503			
2511231054	Pelagibacterium halotolerans B2			
2524614806	Cucumibacter marinus DSM 18995			
2515154079	Martelella mediterranea DSM 17316			
650716042	Hyphomicrobium sp. MC1			
2545555877	Rhizobium selenitireducens ATCC BAA-1503			
648028034	Hyphomicrobium denitrificans ATCC 51888			
637000239	Rhodopseudomonas palustris CGA009			
649633091	Rhodopseudomonas palustris DX-1			
2556921669	Shinella zoogleoides DD12 (SHLA)			
2537561905	Afipia broomeae ATCC 49717			
2537561906	Afipia clevelandensis ATCC 49720			
2537561903	Afipia felis felis ATCC 53690			
637000160	Chelativorans sp. BNC1			
2537562135	Hoeflea phototrophica DFL-43			
640753040	Parvibaculum lavamentivorans DS-1			
638341009	Aurantimonas manganoxydans SI85-9A1			
2522572188	Salinarimonas rosea DSM 21201			
2519899590	Nitratireductor pacificus pht-3B			
2515154196	Meganema perideroedes DSM 15528			
2505119029	Hyphomicrobium sp. VS (Draft1)			
2506783045	Hyphomicrobium denitrificans 1NES1			
2503692000	Hyphomicrobium sulfonivorans S1 (Draft1)			
2521172694	Hyphomicrobium zavarzinii ATCC 27496			
2524614770	Kaistia adipata DSM 17808			

642555153	Rhodopseudomonas palustris TIE-1
648028054	Starkeya novella DSM 506
637000192	Nitrobacter hamburgensis X14
637000193	Nitrobacter winogradskyi Nb-255
2521172615	Kaistia granuli DSM 23481
648276616	Afipia sp. 1NLS2
651324008	Agrobacterium sp. ATCC 31749
2505679039	Aminobacter sp. J15
2508501037	Aminobacter sp. J41
2505679040	Aminobacter sp. J44
2517434012	Ancylobacter sp. FA202
2509276017	Chelativorans sp. J32
2551306370	Chelatococcus sp. GW1
2517434009	Hoeflea sp. 108
2551306656	Methylobacterium sp. B1
2546826724	Methylobacterium sp. EUR3 AL-11
2510065046	Methylocapsa acidiphila B2
643348565	Methylocella silvestris BL2, DSM 15510
650716069	Oligotropha carboxidovorans OM5
2551306133	Pseudaminobacter salicylatoxidans KCT001
2537562120	Rhizobium lupini HPC(L)
2551306670	Rhodopseudomonas sp. B29
2517093040	Xanthobacter sp. 126
2519899517	Xanthobacteraceae bacterium 501b
2531839408	Burkholderia cenocepacia H111
638341037	Burkholderia pseudomallei 1710a

SUPPLEMENTARY TABLE 4. List of over-represented Pfams and criteria used for delineation of "Plant Interaction Determinants" (PID)

Pfam ID	Pfam Name	RNB MEDIAN	NC MEDIAN	Transcript induction in nodule	LPD	% total seqs with SignalP	Antismash Pfam (Seconday metabolite)	PID
**PF00024**	PAN domain	1	0	y	EUK	87.80		PID
**PF00042**	Globin	1	0	y		0.00	y	PID
**PF00078**	Reverse transcriptase (RNA-dependent DNA polymerase)	2	0	y		0.00	y	PID
**PF00080**	Copper/zinc superoxide dismutase (SODC)	1	0	y		87.16		PID
**PF00101**	Ribulose bisphosphate carboxylase, small chain	1	0	y		0.00		PID
**PF00142**	4Fe-4S iron sulfur cluster binding proteins, NifH/frxC family	1	0	y		0.00	y	PID
**PF00194**	Eukaryotic-type carbonic anhydrase	1	0	y		78.70		PID
**PF00232**	Glycosyl hydrolase family 1	1	0	y		16.06	y	PID
**PF00265**	Thymidine kinase	1	0	y		0.00		PID
**PF00450**	Serine carboxypeptidase	1	0		EUK	75.36		PID
**PF00532**	Periplasmic binding proteins and sugar binding domain of LacI family	1	0	y		48.83	y	PID
**PF00614**	Phospholipase D Active site motif	1	0	y		0.00	y	PID
**PF00685**	Sulfotransferase domain (*presumably NodH*)	1	0	y		0.00	y	PID
**PF00703**	Glycosyl hydrolases family 2	1	0	y		0.00	y	PID
**PF00728**	Glycosyl hydrolase family 20, catalytic domain	1	0	y		0.00	y	PID
**PF00743**	Flavin-binding monooxygenase-like	1	0	y		1.26	y	PID
**PF01028**	Eukaryotic DNA topoisomerase I, catalytic core	1	0	y		0.00	y	PID
**PF01219**	Prokaryotic diacylglycerol kinase	1	0	y		0.00	y	PID
**PF01501**	Glycosyl transferase family 8	1	0	y		0.00	y	PID
**PF01610**	Transposase	1	0	y		0.00	y	PID
**PF01663**	Type I phosphodiesterase / nucleotide pyrophosphatase	1	0	y		36.97	y	PID
**PF01928**	CYTH domain	1	0	y		0.00	y	PID
**PF01989**	Protein of unknown function DUF126	1	0	y		0.00		PID
**PF02133**	Permease for cytosine/purines, uracil, thiamine, allantoin	1	0	y		0.00	y	PID
**PF02156**	Glycosyl hydrolase family 26	1	0	y		75.19	y	PID
**PF02230**	Phospholipase/Carboxylesterase	1	0	y		3.88	y	PID
**PF02474**	Nodulation protein A (NodA)	1	0	y	y	0.00		PID
**PF02543**	Carbamoyltransferase	1	0	y		0.00	y	PID
**PF02788**	Ribulose bisphosphate carboxylase large chain, N-terminal domain	1	0	y		0.00		PID
**PF02979**	Nitrile hydratase, alpha chain	1	0	y		0.00	y	PID
**PF03206**	Nitrogen fixation protein NifW	1	0	y		0.00		PID
**PF03264**	NapC/NirT cytochrome c family, N-terminal region	1	0	y		0.00		PID
**PF03270**	Protein of unknown function, DUF269 *(found in Nif-operon)*	1	0	y		0.00		PID
**PF03389**	MobA/MobL family	2	0	y		0.00		PID

**PF03625**	Domain of unknown function DUF302	1	0	y		24.81		PID
**PF03640**	Secreted repeat of unknown function	1	0	y		93.10	y	PID
**PF03775**	Septum formation inhibitor MinC, C-terminal domain	1	0	y		0.00	y	PID
**PF03776**	Septum formation topological specificity factor MinE	1	0	y		0.00	y	PID
**PF03841**	L-seryl-tRNA selenium transferase	1	0	y		0.00	y	PID
**PF03932**	CutC family	1	0	y		0.89		PID
**PF03975**	CheD chemotactic sensory transduction	1	0	y		0.00		PID
**PF04140**	Isoprenylcysteine carboxyl methyltransferase (ICMT) family	1	0	y		0.00	y	PID
**PF04204**	Homoserine O-succinyltransferase	1	0	y		0.00		PID
**PF04209**	homogentisate 1,2-dioxygenase	1	0	y		0.00	y	PID
**PF04214**	Protein of unknown function, DUF	1	0	y		80.73		PID
**PF04227**	Indigoidine synthase A like protein	1	0	y		0.81	y	PID
**PF04234**	CopC domain	1	0	y		75.93	y	PID
**PF04250**	Protein of unknown function (DUF429)	1	0	y		0.00		PID
**PF04285**	Protein of unknown function (DUF444)	1	0	y		0.00		PID
**PF04293**	SpoVR like protein	1	0	y		0.00		PID
**PF04303**	PrpF protein	1	0	y		0.00	y	PID
**PF04319**	NifZ domain	1	0	y		0.00		PID
**PF04392**	ABC transporter substrate binding protein	1	0	y		42.96		PID
**PF04616**	Glycosyl hydrolases family 43	1	0	y		0.00	y	PID
**PF04655**	Aminoglycoside/hydroxyurea antibiotic resistance kinase	1	0	y		0.00	y	PID
**PF04891**	NifQ	1	0	y		0.00		PID
**PF05082**	Rop-like #(found in Nif-operon)	1	0	y		0.00		PID
**PF05114**	Protein of unknown function (DUF692)	1	0	y		0.00	y	PID
**PF05231**	MASE1	1	0	y		0.00	y	PID
**PF05368**	NmrA-like family	2	0	y		0.00	y	PID
**PF05401**	Nodulation protein S (NodS)	1	0	y		0.00		PID
**PF05430**	S-adenosyl-L-methionine-dependent methyltransferase	1	0	y		0.00		PID
**PF05433**	Glycine zipper 2TM domain	1	0	y		80.00		PID
**PF05559**	Protein of unknown function (DUF763)	1	0	y	ARCH	0.00		PID
**PF05610**	Protein of unknown function (DUF779)	1	0	y		0.00	y	PID
**PF05787**	Bacterial protein of unknown function (DUF839)	1	0	y		5.00	y	PID
**PF05962**	HutD	1	0	y		0.00		PID
**PF05992**	SbmA/BacA-like family	1	0	y		0.00		PID
**PF06037**	Bacterial protein of unknown function (DUF922)	2	0		y	65.93		PID
**PF06089**	L-asparaginase II	1	0	y		0.00		PID
**PF06191**	Protein of unknown function (DUF995)	1	0	y	y	71.36		PID
**PF06202**	Amylo-alpha-1,6-glucosidase	1	0	y		0.00		PID
**PF06210**	Protein of unknown function (DUF1003)	1	0	y		0.00		PID

**PF06224**	Winged helix DNA-binding domain	1	0	y		0.00	y	PID
**PF06243**	Phenylacetic acid degradation B	1	0	y		0.00	y	PID
**PF06348**	Protein of unknown function (DUF1059)	1	0	y		0.00		PID
**PF06412**	Conjugal transfer protein TraD	1	0	y		0.00		PID
**PF06441**	Epoxide hydrolase N terminus	2	0	y		19.38	y	PID
**PF06684**	Amino acid synthesis	1	0	y		0.00		PID
**PF06718**	Protein of unknown function (DUF1203)	1	0	y		0.00		PID
**PF06751**	Ethanolamine ammonia lyase large subunit (EutB)	1	0	y		0.00		PID
**PF06772**	Bacterial low temperature requirement A protein (LtrA)	1	0	y		0.00		PID
**PF06792**	Uncharacterised protein family (UPF0261)	1	0	y		0.00		PID
**PF06798**	PrkA serine protein kinase C-terminal domain	1	0	y		0.00		PID
**PF06897**	Protein of unknown function (DUF1269)	1	0	y		0.00		PID
**PF06988**	NifT/FixU protein	1	0	y		0.00		PID
**PF07063**	Domain of unknown function (DUF1338)	1	0	y		0.00		PID
**PF07076**	Protein of unknown function (DUF1344)	1	0	y	y	96.99		PID
**PF07110**	EthD domain	1	0	y		0.00	y	PID
**PF07182**	Protein of unknown function (DUF1402)	1	0		y	93.75		PID
**PF07235**	Protein of unknown function (DUF1427)	1	0	y		0.00		PID
**PF07311**	Dodecin	1	0	y		0.00		PID
**PF07332**	Protein of unknown function (DUF1469)	1	0	y		0.00	y	PID
**PF07336**	Protein of unknown function (DUF1470)	1	0	y		0.00	y	PID
**PF07369**	Protein of unknown function (DUF1488)	1	0	y		0.00		PID
**PF07394**	Protein of unknown function (DUF1501)	1	0	y		37.14		PID
**PF07617**	Protein of unknown function (DUF1579)	1	0	y		0.00	y	PID
**PF07670**	Nucleoside recognition	1	0	y		0.00		PID
**PF07820**	TraC-like protein	1	0	y	y	0.00		PID
**PF07944**	Putative glycosyl hydrolase of unknown function (DUF1680)	1	0	y		0.68	y	PID
**PF07987**	Domain of unkown function (DUF1775)	1	0	y		97.63		PID
**PF07996**	Type IV secretion system proteins	1	0	y		90.91		PID
**PF08003**	Protein of unknown function (DUF1698)	1	0	y		0.00		PID
**PF08013**	Tagatose 6 phosphate kinase	1	0	y		0.00	y	PID
**PF08031**	Berberine and berberine like	1	0	y		0.56	y	PID
**PF08298**	PrkA AAA domain	1	0	y		0.00		PID
**PF08495**	FIST N domain	1	0	y		0.00		PID
**PF08521**	Two-component sensor kinase N-terminal	1	0	y		0.39		PID
**PF08713**	DNA alkylation repair enzyme	1	0	y		0.00	y	PID
**PF08811**	Protein of unknown function (DUF1800)	1	0	y		3.70		PID
**PF08837**	Protein of unknown function (DUF1810)	1	0	y		0.00		PID
**PF08843**	Nucleotidyl transferase of unknown function (DUF1814)	1	0	y		0.00	y	PID

**PF09351**	Domain of unknown function (DUF1993)	1	0	y		0.00		PID
**PF09364**	XFP N-terminal domain	1	0	y		0.00	y	PID
**PF09370**	TIM-barrel signal transduction protein	1	0	y		0.00		PID
**PF09423**	PhoD-like phosphatase	1	0	y		26.03	y	PID
**PF09490**	Probable cobalt transporter subunit (CbtA)	1	0	y		9.60		PID
**PF09587**	Bacterial capsule synthesis protein PGA_cap	1	0	y		7.10	y	PID
**PF09835**	Uncharacterized protein conserved in bacteria (DUF2062)	1	0	y		0.92		PID
**PF09836**	Uncharacterized protein conserved in bacteria (DUF2063)	1	0	y		0.93		PID
**PF09857**	Uncharacterized protein conserved in bacteria (DUF2084)	1	0	y		0.00		PID
**PF09865**	Predicted periplasmic protein (DUF2092)	1	0	y		41.46		PID
**PF09928**	Predicted small integral membrane protein (DUF2160)	1	0	y		0.00		PID
**PF09948**	Predicted metal-binding integral membrane protein (DUF2182)	1	0	y		0.71	y	PID
**PF09992**	Predicted periplasmic protein (DUF2233)	1	0	y		75.21	y	PID
**PF10005**	Uncharacterized protein conserved in bacteria (DUF2248)	1	0	y		0.00		PID
**PF10027**	Predicted integral membrane protein (DUF2269)	1	0	y		0.00	y	PID
**PF10028**	Predicted integral membrane protein (DUF2270)	1	0	y		0.00		PID
**PF10041**	Uncharacterized conserved protein (DUF2277)	1	0	y		0.00		PID
**PF10054**	Predicted periplasmic lipoprotein (DUF2291)	1	0	y		9.09		PID
**PF10095**	Uncharacterized protein conserved in bacteria (DUF2333)	1	0	y		0.00		PID
**PF10101**	Predicted membrane protein (DUF2339)	1	0	y		0.00		PID
**PF10262**	Rdx family	1	0	y		0.00		PID
**PF10423**	Bacterial AMP nucleoside phosphorylase N-terminus	1	0	y		0.00	y	PID
**PF10503**	Esterase PHB depolymerase	1	0	y		11.38	y	PID
**PF10672**	S-adenosylmethionine-dependent methyltransferase	1	0	y		0.00		PID
**PF10931**	Protein of unknown function (DUF2735)	1	0	y	y	0.00		PID
**PF11154**	Protein of unknown function (DUF2934)	3	0	y		0.00		PID
**PF11339**	Protein of unknown function (DUF3141)	1	0	y		0.00		PID
**PF11373**	Protein of unknown function (DUF3175)	1	0	y		0.00		PID
**PF11391**	Protein of unknown function (DUF2798)	1	0	y		0.00		PID
**PF11450**	Protein of unknwon function (DUF3008)	1	0	y	Brucella	0.00		PID
**PF11453**	Protein of unknown function (DUF2950)	1	0	y		73.40		PID
**PF11563**	Protoglobin	1	0	y		0.00		PID
**PF11684**	Protein of unknown function (DUF2380)	1	0		y	83.85		PID
**PF11737**	Protein of unknown function (DUF3300)	1	0	y		73.86		PID
**PF11776**	Domain of unknown function (DUF3315)	1	0	y		98.39		PID
**PF11812**	Domain of unknown function (DUF3333)	1	0	y		0.00		PID
**PF11844**	Domain of unknown function (DUF3364) *(presumably nifK)*	1	0	y		0.00		PID
**PF11845**	Protein of unknown function (DUF3365)	1	0	y		42.86		PID
**PF11899**	Protein of unknown function (DUF3419)	1	0	y		0.00		PID

**PF11927**	Protein of unknown function (DUF3445)	1	0	y		0.00	y	PID
**PF12086**	Protein of unknown function (DUF3563)	1	0	y		0.00		PID
**PF12244**	Protein of unknown function (DUF3606)	2	0	y		0.00		PID
**PF12277**	Protein of unknown function (DUF3618)	1	0	y		0.00	y	PID
**PF12281**	Protein of unknown function (DUF3620)	1	0	y		0.00		PID
**PF12391**	Protocatechuate 3,4-dioxygenase beta subunit N terminal	1	0	y		0.00	y	PID
**PF12464**	Maltose acetyltransferase	1	0	y		0.00	y	PID
**PF12501**	Phosphate ATP-binding cassette transporter	1	0	y		0.00		PID
**PF12573**	2-oxoisovalerate dehydrogenase E1 alpha subunit N terminal	1	0	y		0.00		PID
**PF12893**	Putative lumazine-binding	1	0	y		0.00		PID
**PF12900**	Pyridoxamine 5'-phosphate oxidase	1	0	y		0.00	y	PID
**PF13338**	Domain of unknown function (DUF4095)	1	0	y		0.00		PID
**PF13430**	Domain of unknown function (DUF4112)	1	0	y		0.00		PID
**PF13434**	L-lysine 6-monooxygenase (NADPH-requiring)	1	0	y		0.00	y	PID
**PF13459**	4Fe-4S single cluster domain	1	0	y		0.00	y	PID
**PF13469**	Sulfotransferase family	1	0	y		0.00	y	PID
**PF13474**	SnoaL-like domain	2	0	y		3.64	y	PID
**PF13499**	EF-hand domain pair	1	0	y	EUK	48.65	y	PID
**PF13506**	Glycosyl transferase family 21	1	0	y		5.21	y	PID
**PF13515**	Fusaric acid resistance protein-like	1	0	y		0.00	y	PID
**PF13518**	Helix-turn-helix domain	1	0	y		0.00	y	PID
**PF13577**	SnoaL-like domain	1	0	y		10.53	y	PID
**PF13591**	MerR HTH family regulatory protein	1	0	y		0.00		PID
**PF13619**	KTSC domain	1	0	y		2.51		PID
**PF13657**	HipA N-terminal domain	1	0	y		0.38		PID
**PF13767**	Domain of unknown function (DUF4168)	1	0	y		92.94		PID
**PF13781**	DoxX-like family	1	0	y		0.00		PID
**PF13806**	Rieske-like [2Fe-2S] domain	1	0	y		0.00	y	PID
**PF13823**	Alcohol dehydrogenase GroES-associated	1	0	y		0.00		PID
**PF13936**	Helix-turn-helix domain	1	0	y		0.00	y	PID
**PF14486**	Domain of unknown function (DUF4432)	1	0	y		0.00		PID
**PF14742**	N-terminal domain of (some) glycogen debranching enzymes	1	0	y		0.00		PID
**PF14907**	Uncharacterised nucleotidyltransferase	1	0	y		0.00	y	PID
**PF00089**	Trypsin	1	0			75.16	y	
**PF00148**	Nitrogenase component 1 type Oxidoreductase (*presumably NifD)*	4	0			0.00	y	
**PF00150**	Cellulase (glycosyl hydrolase family 5)	1	0			76.62	y	
**PF00183**	Hsp90 protein	1	0			0.00	y	
**PF00221**	Aromatic amino acid lyase	3	0			3.39	y	
**PF00282**	Pyridoxal-dependent decarboxylase conserved domain	1	0			0.00	y	

**PF00365**	Phosphofructokinase	1	0			1.92	y	
**PF00498**	FHA domain	1	0			0.00	y	
**PF00536**	SAM domain (Sterile alpha motif)	1	0		EUK	0.00		
**PF00585**	C-terminal regulatory domain of Threonine dehydratase	1	0			0.00		
**PF00595**	PDZ domain (Also known as DHR or GLGF)	1	0			75.63		
**PF00668**	Condensation domain	1	0			0.00	y	
**PF00722**	Glycosyl hydrolases family 16	1	0			36.26	y	
**PF00860**	Permease family	1	0			0.00	y	
**PF00872**	Transposase, Mutator family	5	0			0.09	y	
**PF00891**	O-methyltransferase	1	0			0.00	y	
**PF00962**	Adenosine/AMP deaminase	1	0			0.00	y	
**PF01156**	Inosine-uridine preferring nucleoside hydrolase	3	0			1.66	y	
**PF01175**	Urocanase	1	0			0.00		
**PF01183**	Glycosyl hydrolases family 25	3	0			52.88	y	
**PF01232**	Mannitol dehydrogenase Rossmann domain	2	0			0.00		
**PF01244**	Membrane dipeptidase (Peptidase family M19)	1	0			0.00	y	
**PF01276**	Orn/Lys/Arg decarboxylase, major domain	1	0			0.00	y	
**PF01609**	Transposase DDE domain	4	0			0.19	y	
**PF01865**	Protein of unknown function DUF47	1	0			0.00		
**PF01869**	BadF/BadG/BcrA/BcrD ATPase family	1	0			0.00		
**PF01896**	Eukaryotic and archaeal DNA primase small subunit	1	0			0.00		
**PF01963**	TraB family	1	0			3.94		
**PF02056**	Family 4 glycosyl hydrolase	2	0			0.00		
**PF02073**	Thermophilic metalloprotease (M29)	1	0			0.00		
**PF02129**	X-Pro dipeptidyl-peptidase (S15 family)	1	0			3.24	y	
**PF02211**	Nitrile hydratase beta subunit	2	0			0.00	y	
**PF02274**	Amidinotransferase	1	0			0.00	y	
**PF02361**	Cobalt transport protein	1	0			0.00	y	
**PF02436**	Conserved carboxylase domain	1	0			0.00		
**PF02449**	Beta-galactosidase	1	0			0.00	y	
**PF02502**	Ribose/Galactose Isomerase	1	0			0.00	y	
**PF02579**	Dinitrogenase iron-molybdenum cofactor	2	0			0.00		
**PF02588**	Uncharacterized BCR, YitT family COG1284	1	0			0.00		
**PF02614**	Glucuronate isomerase	1	0			0.00		
**PF02624**	YcaO-like family	2	0			0.00	y	
**PF02668**	Taurine catabolism dioxygenase TauD, TfdA family	1	0			0.00	y	
**PF02690**	Na+/Pi-cotransporter	1	0			0.00		
**PF02733**	Dak1 domain	3	0			0.00	y	
**PF02734**	DAK2 domain	3	0			0.50	y	

**PF02743**	Cache domain	3	0			2.42	y	
**PF02796**	Helix-turn-helix domain of resolvase	1	0			0.00		
**PF02805**	Metal binding domain of Ada	1	0			0.00	y	
**PF02836**	Glycosyl hydrolases family 2, TIM barrel domain	1	0			0.00	y	
**PF02837**	Glycosyl hydrolases family 2, sugar binding domain	2	0			0.00	y	
**PF02909**	Tetracyclin repressor, C-terminal all-alpha domain	1	0			0.00	y	
**PF03050**	Transposase IS66 family	7	0			0.00	y	
**PF03180**	NLPA lipoprotein	1	0			84.25	y	
**PF03412**	Peptidase C39 family	1	0			6.93	y	
**PF03428**	Replication protein C N-terminal domain	2	0		y	0.00		
**PF03479**	Domain of unknown function (DUF296)	1	0			2.40		
**PF03594**	Benzoate membrane transport protein	1	0			0.00	y	
**PF03681**	Uncharacterised protein family (UPF0150)	1	0			0.00		
**PF03693**	Uncharacterised protein family (UPF0156)	3	0			0.00		
**PF03704**	Bacterial transcriptional activator domain	1	0			0.00	y	
**PF03709**	Orn/Lys/Arg decarboxylase, N-terminal domain	1	0			0.00	y	
**PF03711**	Orn/Lys/Arg decarboxylase, C-terminal domain	1	0			0.00	y	
**PF03779**	SPW repeat	1	0			0.00		
**PF03786**	D-mannonate dehydratase (UxuA)	1	0			0.00	y	
**PF03894**	D-xylulose 5-phosphate/D-fructose 6-phosphate phosphoketolase	1	0			0.00	y	
**PF03922**	OmpW family	1	0			88.11		
**PF03924**	CHASE domain	1	0			2.26		
**PF04012**	PspA/IM30 family	1	0			0.00		
**PF04120**	Low affinity iron permease	1	0			0.00	y	
**PF04134**	Protein of unknown function, DUF393	1	0			0.00	y	
**PF04198**	Putative sugar-binding domain	5	0			0.00	y	
**PF04230**	Polysaccharide pyruvyl transferase	2	0			0.00	y	
**PF04237**	YjbR	1	0			0.00	y	
**PF04239**	Protein of unknown function (DUF421)	1	0			0.00	y	
**PF04263**	Thiamin pyrophosphokinase, catalytic domain	1	0			0.97		
**PF04265**	Thiamin pyrophosphokinase, vitamin B1 binding domain	1	0			1.09		
**PF04299**	Putative FMN-binding domain	1	0			0.00	y	
**PF04305**	Protein of unknown function (DUF455)	1	0			0.00		
**PF04332**	Protein of unknown function (DUF475)	1	0			0.00	y	
**PF04397**	LytTr DNA-binding domain	1	0			0.00	y	
**PF04412**	Protein of unknown function (DUF521)	1	0		EUK	0.00		
**PF04444**	Catechol dioxygenase N terminus	2	0			0.00	y	
**PF04463**	Protein of unknown function (DUF523)	1	0			0.00		
**PF04471**	Restriction endonuclease	1	0			0.00	y	

**PF04717**	Phage-related baseplate assembly protein	1	0			0.00	y	
**PF04892**	VanZ like family	1	0			2.96	y	
**PF04952**	Succinylglutamate desuccinylase / Aspartoacylase family	2	0			1.25		
**PF04954**	Siderophore-interacting protein	1	0			0.00	y	
**PF04962**	KduI/IolB family	2	0			0.00		
**PF04965**	Gene 25-like lysozyme	1	0			0.00	y	
**PF04982**	HPP family	1	0			0.00	y	
**PF05014**	Nucleoside 2-deoxyribosyltransferase	1	0			0.00	y	
**PF05025**	RbsD / FucU transport protein family	2	0			0.00		
**PF05035**	2-keto-3-deoxy-galactonokinase	1	0			0.00		
**PF05088**	Bacterial NAD-glutamate dehydrogenase	1	0			0.00		
**PF05138**	Phenylacetic acid catabolic protein	2	0			0.00	y	
**PF05139**	Erythromycin esterase	1	0			0.00	y	
**PF05171**	Haemin-degrading HemS.ChuX domain	1	0			0.00		
**PF05228**	CHASE4 domain	1	0			0.90	y	
**PF05336**	Domain of unknown function (DUF718)	1	0			0.00		
**PF05360**	yiaA/B two helix domain	1	0			0.00		
**PF05406**	WGR domain	1	0			0.00	y	
**PF05425**	Copper resistance protein D	1	0			26.79		
**PF05544**	Proline racemase	2	0			0.28		
**PF05598**	Transposase domain (DUF772)	3	0			0.00	y	
**PF05717**	IS66 Orf2 like protein	5	0			0.00	y	
**PF05954**	Phage late control gene D protein (GPD)	1	0			0.00	y	
**PF05973**	Phage derived protein Gp49-like (DUF891)	1	0			0.00		
**PF05985**	Ethanolamine ammonia-lyase light chain (EutC)	1	0			0.00		
**PF05987**	Bacterial protein of unknown function (DUF898)	1	0			0.00		
**PF05988**	Bacterial protein of unknown function (DUF899)	4	0			0.00	y	
**PF06055**	Exopolysaccharide synthesis, ExoD	1	0			0.00		
**PF06057**	Bacterial virulence protein (VirJ)	1	0			90.35	y	
**PF06165**	Glycosyltransferase family 36	1	0			0.00		
**PF06169**	Protein of unknown function (DUF982)	9	0		y	0.00		
**PF06187**	Protein of unknown function (DUF993)	1	0			0.00		
**PF06204**	Putative carbohydrate binding domain	1	0			0.00		
**PF06205**	Glycosyltransferase 36 associated family	1	0			0.00		
**PF06267**	Family of unknown function (DUF1028)	1	0			13.04		
**PF06276**	Ferric iron reductase FhuF-like transporter	1	0			0.00	y	
**PF06283**	Trehalose utilisation	2	0			0.00		
**PF06314**	Acetoacetate decarboxylase (ADC)	1	0			0.00	y	
**PF06455**	NADH dehydrogenase subunit 5 C-terminus	1	0			0.00		

**PF06481**	COX Aromatic Rich Motif	1	0			1.01		
**PF06537**	Protein of unknown function (DUF1111)	1	0			69.57		
**PF06719**	AraC-type transcriptional regulator N-terminus	2	0			0.62		
**PF06745**	KaiC	1	0			0.00		
**PF06779**	Protein of unknown function (DUF1228)	1	0			0.00		
**PF06906**	Protein of unknown function (DUF1272)	1	0			0.00		
**PF06964**	Alpha-L-arabinofuranosidase C-terminus	1	0			0.00		
**PF07005**	Protein of unknown function, DUF1537	3	0			0.00		
**PF07040**	Protein of unknown function (DUF1326)	1	0			0.00	y	
**PF07081**	Protein of unknown function (DUF1349)	1	0			0.00		
**PF07171**	MlrC C-terminus	2	0			0.00		
**PF07221**	N-acylglucosamine 2-epimerase (GlcNAc 2-epimerase)	1	0			0.00		
**PF07228**	Stage II sporulation protein E (SpoIIE)	1	0			0.00	y	
**PF07364**	Protein of unknown function (DUF1485)	2	0			0.00		
**PF07366**	SnoaL-like polyketide cyclase	2	0			8.50	y	
**PF07433**	Protein of unknown function (DUF1513)	1	0			34.58		
**PF07506**	RepB plasmid partitioning protein	4	0			0.00		
**PF07582**	AP endonuclease family 2 C terminus	1	0			0.00		
**PF07704**	Rv0623-like transcription factor	1	0			0.00		
**PF07756**	Protein of unknown function (DUF1612)	1	0		y	0.00		
**PF07786**	Protein of unknown function (DUF1624)	1	0			0.00	y	
**PF07804**	HipA-like C-terminal domain	2	0			0.36		
**PF07805**	HipA-like N-terminal domain	1	0			0.36		
**PF07812**	TfuA-like protein	1	0		y	0.00	y	
**PF07848**	PaaX-like protein	1	0			0.00	y	
**PF07876**	Stress responsive A/B Barrel Domain	2	0			0.00	y	
**PF07931**	Chloramphenicol phosphotransferase-like protein	1	0			0.00		
**PF08002**	Protein of unknown function (DUF1697)	2	0			0.00	y	
**PF08009**	CDP-alcohol phosphatidyltransferase 2	1	0			0.00		
**PF08021**	Siderophore-interacting FAD-binding domain	1	0			0.00	y	
**PF08125**	Mannitol dehydrogenase C-terminal domain	2	0			0.00		
**PF08223**	PaaX-like protein C-terminal domain	1	0			0.00	y	
**PF08269**	Cache domain	3	0			7.85		
**PF08291**	Peptidase M15	1	0			58.72		
**PF08362**	YcdC-like protein, C-terminal region	1	0			0.00		
**PF08388**	Group II intron, maturase-specific domain	1	0			0.00	y	
**PF08410**	Domain of unknown function (DUF1737)	1	0			0.00		
**PF08421**	Putative zinc binding domain	1	0			0.00	y	
**PF08479**	POTRA domain, ShlB-type	1	0			58.64		

**PF08483**	IstB-like ATP binding N-terminal	1	0			0.00		
**PF08484**	C-methyltransferase C-terminal domain	1	0			0.00	y	
**PF08530**	X-Pro dipeptidyl-peptidase C-terminal non-catalytic domain	1	0			1.66	y	
**PF08532**	Beta-galactosidase trimerisation domain	1	0			0.00	y	
**PF08548**	Peptidase M10 serralysin C terminal	1	0			0.00	y	
**PF08592**	Domain of unknown function (DUF1772)	1	0			0.00	y	
**PF08681**	Protein of unknown function (DUF1778)	1	0			0.00	y	
**PF08734**	GYD domain	2	0			0.00		
**PF09130**	Domain of unknown function (DUF1932)	1	0			0.00		
**PF09209**	Domain of unknown function (DUF1956)	1	0			0.00	y	
**PF09347**	Domain of unknown function (DUF1989)	1	0			0.00	y	
**PF09363**	XFP C-terminal domain	1	0			0.00	y	
**PF09383**	NIL domain	1	0			0.00	y	
**PF09391**	Protein of unknown function (DUF2000)	1	0			0.00		
**PF09411**	Lipid A 3-O-deacylase (PagL)	1	0			72.73		
**PF09604**	F subunit of K+-transporting ATPase (Potass_KdpF)	1	0			0.00		
**PF09678**	Cytochrome c oxidase caa3 assembly factor (Caa3_CtaG)	1	0			22.64	y	
**PF09828**	Chromate resistance exported protein	1	0			0.00		
**PF09863**	Uncharacterized protein conserved in bacteria (DUF2090)	1	0			0.00		
**PF09864**	Membrane-bound lysozyme-inhibitor of c-type lysozyme	1	0			57.61		
**PF09924**	Uncharacterized conserved protein (DUF2156)	1	0			0.00	y	
**PF09929**	Uncharacterized conserved protein (DUF2161)	1	0			0.00		
**PF09939**	Uncharacterized protein conserved in bacteria (DUF2171)	1	0			0.00		
**PF09972**	Predicted membrane protein (DUF2207)	1	0			76.32		
**PF09976**	Tetratricopeptide repeat	1	0			0.00		
**PF09977**	Putative Tad-like Flp pilus-assembly	1	0			0.00		
**PF09981**	Uncharacterized protein conserved in bacteria (DUF2218)	1	0			0.00		
**PF09990**	Predicted membrane protein (DUF2231)	1	0			0.00	y	
**PF10000**	ACT domain	1	0			0.00		
**PF10016**	Predicted secreted protein (DUF2259)	1	0			86.87		
**PF10022**	Uncharacterized protein conserved in bacteria (DUF2264)	1	0			0.00	y	
**PF10025**	Uncharacterized conserved protein (DUF2267)	1	0			0.00	y	
**PF10048**	Predicted integral membrane protein (DUF2282)	1	0			100.00		
**PF10087**	Uncharacterized protein conserved in bacteria (DUF2325)	1	0			0.00		
**PF10091**	Putative glucoamylase	2	0			0.37		
**PF10115**	Transcriptional activator HlyU	1	0			0.00		
**PF10442**	FIST C domain	1	0			0.00		
**PF10755**	Protein of unknown function (DUF2585)	1	0		y	0.00		
**PF11294**	Protein of unknown function (DUF3095)	1	0			0.00		

**PF11329**	Protein of unknown function (DUF3131)	1	0			1.09		
**PF11512**	Agrobacterium tumefaciens protein Atu4866	1	0			31.82	y	
**PF11604**	Copper binding periplasmic protein CusF	1	0			95.40		
**PF11706**	CGNR zinc finger	1	0			0.00	y	
**PF11798**	IMS family HHH motif	1	0			0.00	y	
**PF11800**	Replication protein C C-terminal region	3	0		y	0.00		
**PF11896**	Domain of unknown function (DUF3416)	1	0			0.00		
**PF11972**	HTH DNA binding domain	1	0		y	0.00		
**PF11975**	Family 4 glycosyl hydrolase C-terminal domain	2	0			0.00		
**PF12200**	Domain of unknown function (DUF3597)	1	0			22.12		
**PF12411**	Choline sulfatase enzyme C terminal	1	0			0.00		
**PF12536**	Patatin phospholipase	1	0			0.00		
**PF12708**	Pectate lyase superfamily protein	1	0			30.08	y	
**PF12903**	Protein of unknown function (DUF3830)	1	0			0.00		
**PF13005**	zinc-finger binding domain of transposase IS66	4	0			0.00	y	
**PF13007**	Transposase C of IS166 homeodomain	4	0			0.00	y	
**PF13087**	AAA domain	1	0			0.00		
**PF13088**	BNR repeat-like domain	1	0			8.76	y	
**PF13230**	Glutamine amidotransferases class-II	1	0			0.00		
**PF13249**	Prenyltransferase-like	1	0			0.00	y	
**PF13358**	DDE superfamily endonuclease	4	0			0.00	y	
**PF13382**	Adenine deaminase C-terminal domain	2	0			0.00		
**PF13384**	Homeodomain-like domain	1	0			0.00	y	
**PF13396**	Phospholipase_D-nuclease N-terminal	1	0			0.95	y	
**PF13452**	N-terminal half of MaoC dehydratase	1	0			0.00	y	
**PF13454**	FAD-NAD(P)-binding	2	0			0.00	y	
**PF13488**	Glycine zipper	3	0			59.38		
**PF13495**	Phage integrase, N-terminal SAM-like domain	1	0			0.00	y	
**PF13523**	Acetyltransferase (GNAT) domain	1	0			0.00	y	
**PF13524**	Glycosyl transferases group 1	3	0			0.00	y	
**PF13528**	Glycosyl transferase family 1	2	0			0.49	y	
**PF13536**	Multidrug resistance efflux transporter	1	0			0.00	y	
**PF13551**	Winged helix-turn helix	5	0			0.00	y	
**PF13564**	DoxX-like family	1	0			0.00	y	
**PF13566**	Domain of unknown function (DUF4130	1	0			0.00		
**PF13586**	Transposase DDE domain	2	0			0.00	y	
**PF13587**	N-terminal domain of DJ-1_PfpI family	1	0			0.47	y	
**PF13593**	SBF-like CPA transporter family (DUF4137)	1	0			2.44		
**PF13594**	Amidohydrolase	1	0			1.75	y	

**PF13596**	PAS domain	1	0			0.00	y	
**PF13610**	DDE domain	1	0			0.00	y	
**PF13649**	Methyltransferase domain	1	0			0.00	y	
**PF13672**	Protein phosphatase 2C	1	0			0.00		
**PF13704**	Glycosyl transferase family 2	1	0			0.00		
**PF13744**	Helix-turn-helix domain	1	0			0.00		
**PF13751**	Transposase DDE domain	1	0			0.18	y	
**PF13817**	IS66 C-terminal element	5	0			0.00	y	
**PF13844**	Glycosyl transferase family 41	1	0			0.00		
**PF14552**	Tautomerase enzyme	1	0			0.00		
**PF14696**	Hydroxyphenylpyruvate dioxygenase, HPPD, N-terminal	2	0			0.00	y	
**PF14697**	4Fe-4S dicluster domain	1	0			0.00	y	
**PF14707**	C-terminal region of aryl-sulfatase	1	0			55.95		

RNB median is the median gene count for the corresponding Pfam domain from 163 RNB genomes

NC Median is the median gene count from 69 phylogenetically related control genomes

Transcript induction in nodule column is derived from significant results from previous transcriptomic studies referenced in the manuscript

LPD is "limited phylogenetic distribution" as described in the manuscript.

"% total sequences with SignalP" is the proportion of sequences bearing a singal peptide motif for potential secretion

"Y" indicates Pfam domains used by the AntiSMASH program to predict biosynthetic clusters, highlighting putative secondary metabolite functions.

PID is "plant interaction determinants" as described in the manuscript.

Green highlights of selected cells is to draw attention to "positive control" functions involved in nodulation or nitrogen fixation

Supplementary Table 5. List of "enriched" Pfams based on statistical analysis of pairwise comparisons of gene counts between RNB and NC genomes.

"Enriched" Pfam ID (based on pairwise comparisons)	Pfam Name	# of significantly different pairs	RNB Q1 (25th %)	NC Q3 (75th %)	RNB Median	NC Median
PF00211	Adenylate and Guanylate cyclase catalytic domain	7,734	15	6	27	2
PF03466	LysR substrate binding domain	7,226	78	46	90	30
PF00126	Bacterial regulatory helix-turn-helix protein, lysR family	7,105	78	46	90	30
PF13407	Periplasmic binding protein domain	6,995	13	10	31	3
PF00528	Binding-protein-dependent transport system IM component	6,584	126	120	175	62
PF00356	Bacterial regulatory proteins, lacI family	6,020	7	8	25	3
PF13377	Periplasmic binding protein-like domain	5,423	6	8	20	3
PF06169	Protein of unknown function (DUF982)	5,310	0	0	9	0
PF01547	Bacterial extracellular solute-binding protein	5,133	7	6	15	1
PF00392	GntR-like bacterial transcription factors	5,116	39	35	51	20
PF12833	Helix-turn-helix	5,073	36	23	43	16
PF01261	Xylose isomerase-like TIM barrel	4,812	20	23	28	10
PF08352	Oligopeptide/dipeptide transporter, C-terminal region	4,810	9	8	21	3
PF00005	ABC transporter	4,772	187	157	208	120
PF01408	Oxidoreductase family, NAD-binding Rossmann fold	4,718	10	11	24	5
PF07729	FCD domain	4,685	27	25	35	13
PF08402	TOBE domain	4,562	17	18	32	8
PF02653	Branched-chain amino acid transport system / permease component	4,113	56	44	65	35
PF13416	Bacterial extracellular solute-binding protein	4,091	16	13	26	7
